# Exploring the Co-Crystallization Landscape of One-Dimensional Coordination Polymers Using a Molecular Electrostatic Potential-Driven Approach

**DOI:** 10.1021/acs.cgd.3c00615

**Published:** 2023-09-01

**Authors:** Ozana Mišura, Ivan Kodrin, Mladen Borovina, Mateja Pisačić, Viraj De Silva, Christer B. Aakeröy, Marijana Đaković

**Affiliations:** † Department of Chemistry, Faculty of Science, University of Zagreb, Horvatovac 102a, Zagreb 10000, Croatia; ‡ Department of Chemistry, Kansas State University, Manhattan, Kansas 66506-0401, United States

## Abstract

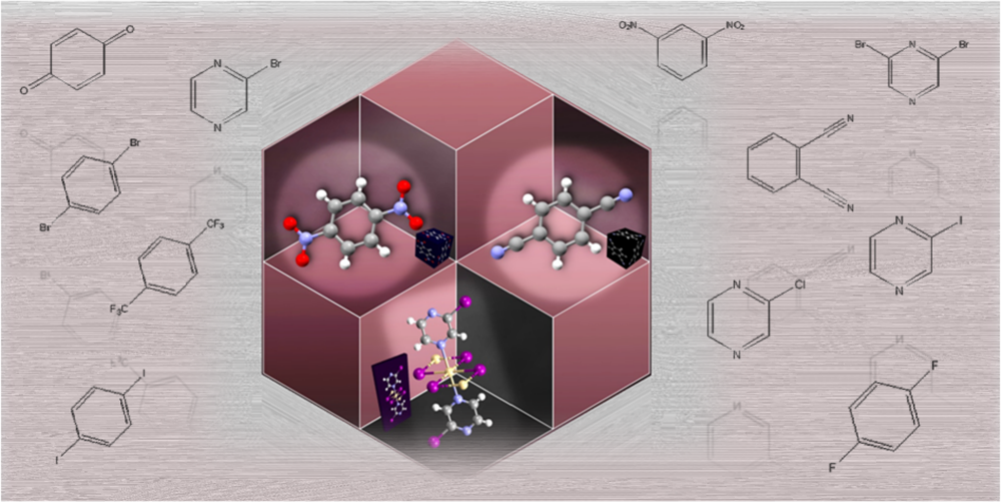

The ability of coordination polymers (CPs) to form multicomponent heteromeric materials, where the key structural features of the parent CP are retained, has been explored via molecular electrostatic potential-driven co-crystallization technologies. Thirteen co-formers presenting hydrogen-bond donors activated through a variety of electron-withdrawing functionalities were employed, and the extent of activation was evaluated using molecular electrostatic potential values. Attempted co-crystallizations of the seven most promising co-formers with a family of nine CPs ([CdX′_2_(X-pz)_2_]_*n*_; X′ = I, Br, and Cl; X = I, Br, and Cl) resulted in six successful outcomes; all four of the structurally characterized compounds displayed the intended hydrogen bond. The rationalization of the main structural features revealed that strict structural and electrostatic requirements were imposed on effective co-formers; only co-formers with highly activated hydrogen-bond donors, and with a 1,4-orientation of electron-withdrawing moieties bearing effective acceptor sites, were successfully implemented into the three-dimensional architectures composed of one-dimensional building units of CPs.

## Abstract 2

The boundaries on the synthetic landscape where co-crystals of coordination polymers and organic co-formers can be reliably delivered were explored based on calculated molecular electrostatic potentials (MEPs). A combination of MEP values and surface site interaction point pairing energies showed as a useful tool for narrowing down the experimental search space.

## Introduction

Chemical synthesis is a well-established scientific discipline, and thanks to many reliable and carefully optimized named reactions, we are today capable of creating molecules of exceptional structural complexities.^1,2^

Unfortunately, the lack of reliable bottom-up approaches to the design and synthesis of new solids with predictable or tunable bulk *properties* remains one of the biggest challenges for current materials science.^3^ At the core of this problem lies the fact that structure governs function, and since universal crystal-structure prediction remains a largely intractable problem, genuine property design is still elusive. Because control over the three-dimensional crystal structure is a necessary requirement for accurate bottom-up design, one way to simplify the challenge is to begin with a specific crystal structure and then gradually alter the chemical composition of the material in a modular manner without changing the overall crystal packing. This strategy has been expressed by organic solid-state chemists using co-crystallization technologies wherein a homomeric solid is combined with a “co-former” in such a way that the chemical composition changes without drastic alterations to the main structural features of the parent.^4,5^ In this way, several bulk properties have been altered in a systematic manner, e.g., thermal stability, aqueous solubility, impact sensitivity, or mechanical properties, to name a few.^6−11^

Effective approaches for altering physical properties of metals and coordination complexes have a long history. In metallurgy, the partial replacement of one metal for another, without substantially altering the crystal packing or structure, has delivered stainless steel, brass, and many other alloys.^12^ The degree of “doping” can be directly related to specific changes in physical properties, simply because the crystal structure of the new material does not deviate from that of the parent.^13,14^ In coordination chemistry, there are opportunities for modular changes to chemical composition, without unwanted changes to the overall crystal structure. For example, a six-coordinate M(II) metal ion can be replaced with another divalent metal ion with the same geometric requirement without altering the structure,^15,16^ but photophysical and magnetic properties may be substantially altered.^17^ Counter ions can also be replaced without unwanted structural changes, and there are numerous examples of, e.g., chloride/bromide pairs of transition-metal complexes that are isostructural.^18−20^

However, the reports on systematic structure–function exploration where a “co-former” is incorporated into a crystalline coordination polymer (CP) with the objectives of (i) retaining key structural features of the parent and (ii) finding guidelines for which type of co-former is most likely to fit seamlessly into the new lattice are scarce.^21^ Identifying effective co-formers often requires extensive experimental screening, but it would be very valuable if the experimental search space could be narrowed *a priori* via a simple computational approach.^22,23^

To seek a reliable link between co-crystal synthesis and solid-state coordination chemistry, we decided to examine if a series of compounds with distinct and well-defined mechanical properties could be used as a proof-of-principle exploration. They all contain one-dimensional (1-D) cadmium(II)-based structural “spine”, but differences in intermolecular interactions produce strikingly different mechanical responses, from almost inelastic to modestly and extensively elastic.^24^ Furthermore, it has been shown that the incorporation of small symmetric co-formers capable of strengthening a specific supramolecular link successfully enhances the elastic behavior of the parent CP crystal.^25^

**Scheme 1:**
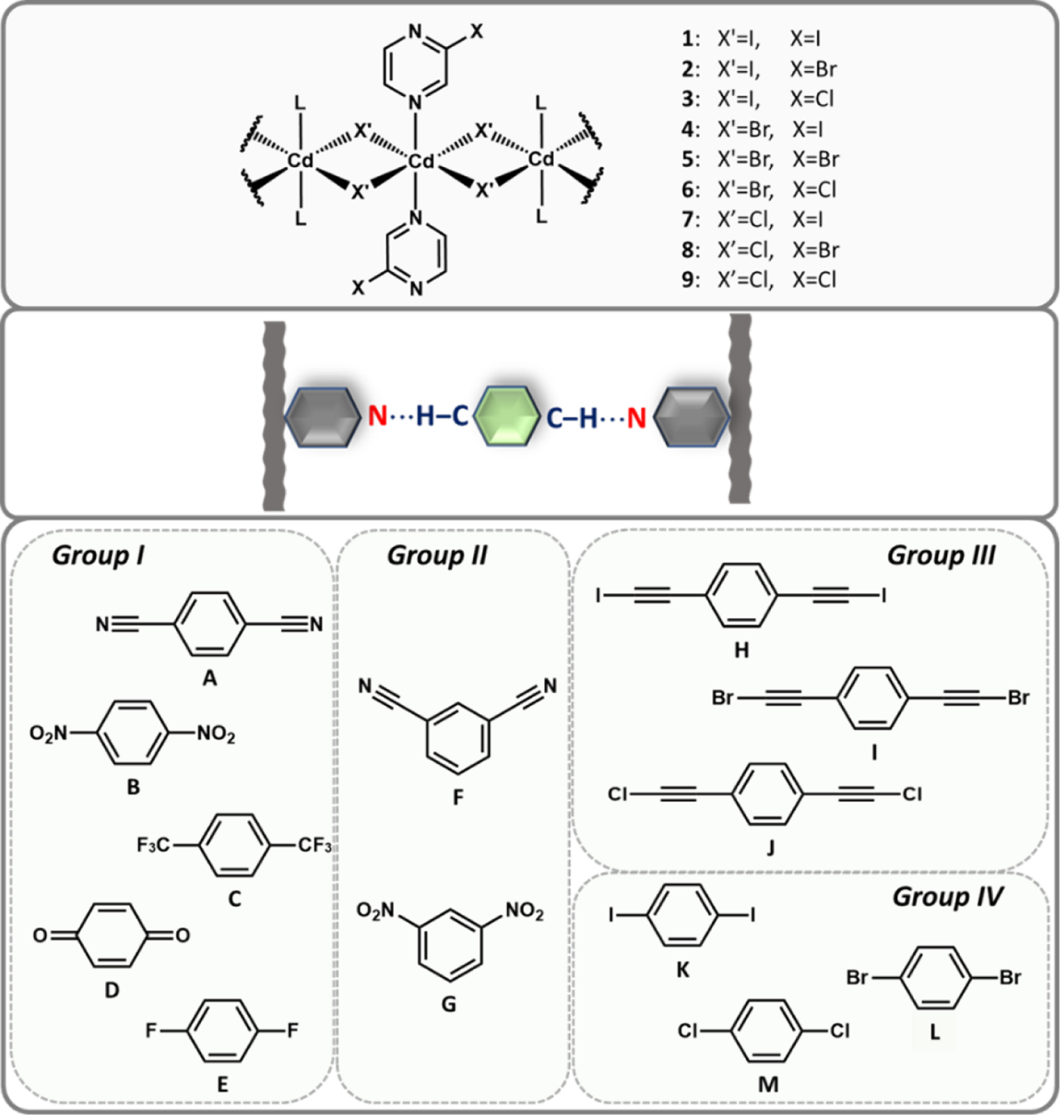
Starting 1-D CPs (**1**–**9**; Top) and Organic Co-Formers (**A–M**; Down) Used in Co-Crystallization Reactions to Acquire the Targeted C–H···N Link (Middle)

In this study, we opted for a larger library of both coordination compounds and co-formers to provide a more diverse data set for systematic exploration of both electrostatic *and* steric influences in the synthesis of targeted CP-based co-crystals (Scheme 1).^25^ The selected CPs comprise all nine possible combinations of cadmium(II) halides with halopyrazines (CdX′_2_, X′ = I, Br, and Cl; X-pz, X = I, Br, and Cl), while the co-former requirements lead us to small, symmetric, single-aromatic-ring molecules presenting “activated” hydrogen atoms for establishing specific hydrogen bonds with the parent (Scheme 1). The activation of co-former hydrogen atoms was achieved by equipping the co-formers with two electron-withdrawing moieties.

The co-formers were organized into four groups according to their geometric characteristic and the availability of additional donor/acceptor sites residing at the electron-withdrawing arms. While *group I* and *group II* both presented hydrogen-bond acceptor sites but with different relative arrangements of the electron-withdrawing moieties (*group I*, 1,4-orientation; *group II*, 1,3-orientation), *group III* and *group IV* were bearing halogen-bond donors on the arms of different lengths (*group III*, long arms; *group IV*, short arms).

For all the CPs and co-formers, molecular electrostatic potential (MEP) at hydrogen- and halogen-bond donor and acceptor sites was calculated as MEP has already proven a useful tool in planning co-crystal synthesis.^26^ The computational work was accompanied by synthetic and structural efforts, and the results were rationalized based on the calculated MEP values and surface site interaction point pairing energies.^27^

## Results and Discussion

### MEP-Based Strategy Plan

**Figure 1:**
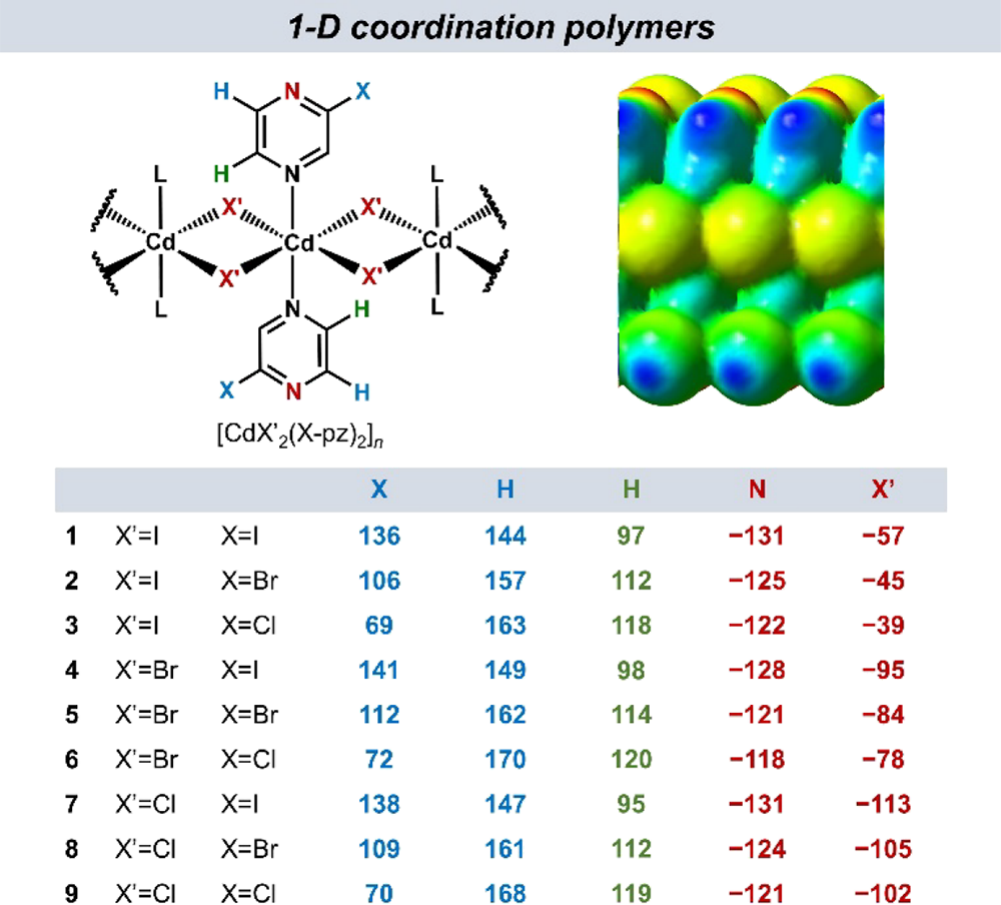
Calculated MEP values (in kJ mol^–1^ e^–1^) for the starting 1-D CPs **1**–**9**. Geometries optimized at the PBE-D3/pob-TZVP-rev2 level of theory.

**Figure 2:**
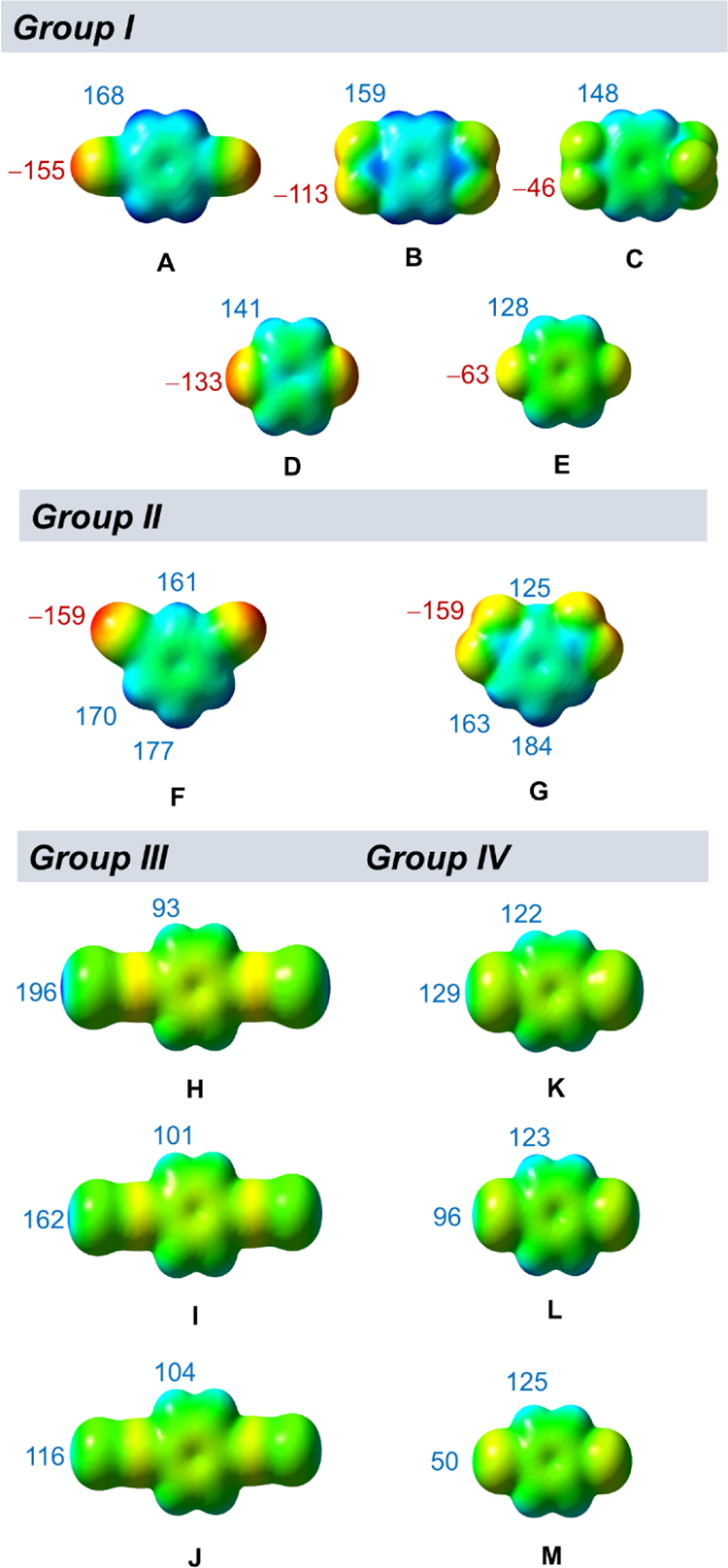
Selected co-formers and calculated MEP values (in kJ mol^–1^ e^–1^). Geometries optimized at the PBE-D3/pob-TZVP-rev2 level of theory.

Calculated MEP values were used to rank the hydrogen-bond donor and acceptor sites of both CPs and co-formers; a more positive MEP value equates to a better hydrogen-bond donor, whereas a more negative value indicates a better hydrogen-bond acceptor. The calculated MEP values for the CPs and co-formers are listed in Figures 1 and 2, respectively.

For the nine CPs (**1**–**9**), two regions on the molecular surfaces with the highest (X and H atoms on the pz ligand) and the lowest electrostatic potential values (non-coordinated pyrazine N atom and bridging halide, X') were identified (Figure 1). In all CPs, the non-coordinated pyrazine nitrogen atom was the most negative site, with the iodo- (**1**, **4**, and **7**) and chloropyrazine derivatives (**3**, **6**, and **9**) populating two opposite ends of the MEP range (i.e., having the most and least negative MEP values, respectively). Moreover, the nitrogen atom MEP values spanned a relatively small range across the CP group without any noticeable separations between them (**1**–**9**) that would not offer a basis for narrowing down the initial set of CPs. Consequently, all nine CPs were taken into the experimental co-crystal screening.

On the other hand, MEPs for the co-formers displayed a much larger range, which led us to limit the initial set of co-formers to only the most promising ones from each group, *groups I*–*IV* (Figure 2)*.*

The members of *group I* presented a wide range of MEP values associated with the “activated” hydrogen atoms, and thus, only co-formers with the largest positive MEP values were selected (**A**, **B**, and **C**). On the other hand, *group II* included only two co-formers, the only examples of 1,3-isomers; therefore, both co-formers (**F** and **G**) were used in the experimental co-crystal screen.

In contrast, co-formers of *groups III and IV* showed only slight differences in the H-atom MEP values. Since these co-formers were additionally equipped with halogen-bond donor sites at the electron-withdrawing moieties, solely iodine derivatives (**H** and **K**), as the best halogen-bond donors, were selected for the co-crystal screen.

### Co-Crystal Synthesis

**Scheme 2:**
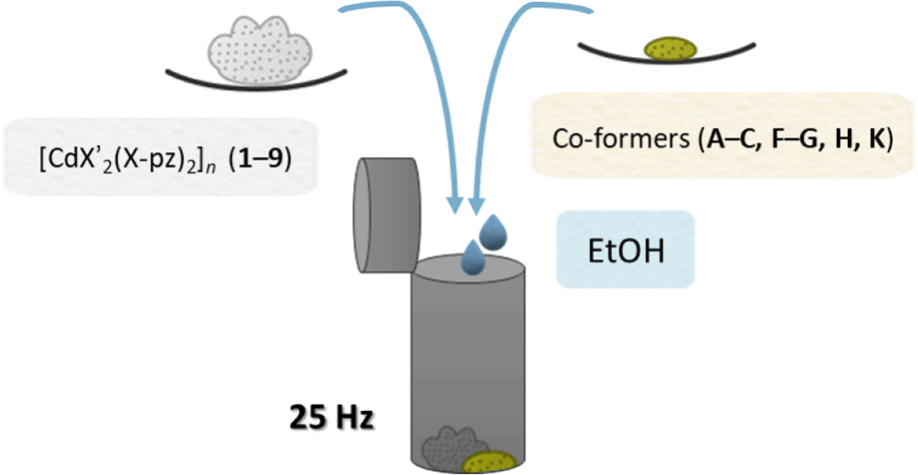
Solvent-Assisted Grinding Employed as a Synthetic Method for Co-Crystallizing CPs (**1**–**9**) with Selected Small Organic Co-Formers (**A**–**C**, **F**, **G**, **H**, and **K**)

The co-crystal syntheses were performed via solvent-assisted grinding, a proven and commonly used approach.^28−30^ Seven co-formers (**A**, **B**, **C**, **F**, **G**, **H**, and **K**) were put through a co-crystal screen against nine CPs (**1**–**9**) in a 1:1 stoichiometric ratio and with a few drops of solvent (Scheme 2). Powder X-ray diffraction (PXRD) was used to prescreen the attempted co-crystallizations, and bulk products with PXRD patterns displaying new peak(s) below 10° (2θ) were identified as successes. Out of 63 co-crystallization combinations, according to the PXRD patterns, only four new co-crystalline forms were obtained (**2:A**, **3:A**, **5:A**, and **6:A**), which with two previously reported ones (**1:A** and **1:B**)^25^ left us with a relatively small set of co-crystals (**1:A**, **1:B**, **2:A**, **3:A**, **5:A**, and **6:A**) for further exploration.

### Structural Study

To obtain X-ray-quality single crystals, bulk solids from successful grinding experiments were transferred to small vials, dissolved in a variety of solvents at room temperature, and left undisturbed to allow the solvent to slowly evaporate. Crystals of **1:A**, **1:B**, **2:A**, and **5:A** were successfully grown from methanol, acetone, or acetonitrile, while the efforts to get single crystals of **3:A** and **6:A** from the same solvents failed (see the Supporting Information (SI)). Although being successfully formed via solvent-assisted grinding, upon dissolution, they disintegrated and yielded a mixture of single-component crystals of starting substances, **3** and **A** and **6** and **A**. This underlines the importance of solvent, solubility of the two components, and nucleation kinetics on the outcome of crystallization products irrespective of previous co-crystal formation via mechanochemical synthesis. In short, the process for obtaining co-crystals suitable for single-crystal X-ray diffraction (SCXRD) from solution is not always straightforward and may require systematic and detailed optimization of crystallization procedures and methods. The low-temperature SCXRD data collection was then performed for **1:A**, **1:B**, **2:A**, and **5:A** (Tables S1–S6).

**Figure 3:**
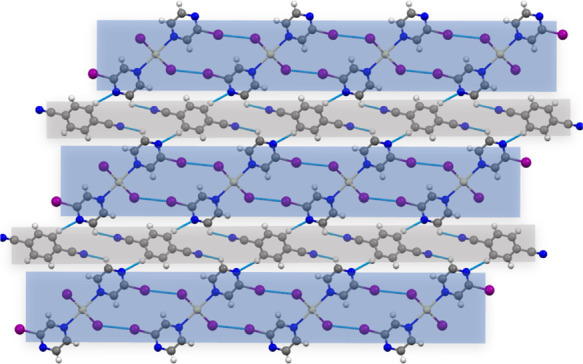
Alternation of 2-D layers of **1** and **A** in the crystal packing of **1:A**.^25^ Arrangement of 2-D layers in **1:B**, **2:A**, and **5:A** is analogous to that in **1:A**.

The low-temperature crystal structure of **1:A** did not reveal any substantial difference in comparison with the previously reported room-temperature structure (Figure 3).^25^ The 2-D layers observed in **1**, being composed of 1-D CP building units and dibridged by complementary C–I···I_Cd_ halogen bonds, remained preserved in **1:A**. The co-former molecules (**A**) just intervened between the neighboring layers, thus forming an alternating 2-D-layer arrangement of the parent (**1**) and co-former (**A**). Each co-former links two polymeric units from adjacent 2-D layers via two C_A_–H···N_CP_ hydrogen bonds and is additionally anchored by two hydrogen bonds of the C_CP_–H···N_A_ type involving neighboring CP units from the same 2-D layers. Thus, each co-former molecule spans four neighboring polymeric units from two 2-D layers.

**Figure 4:**
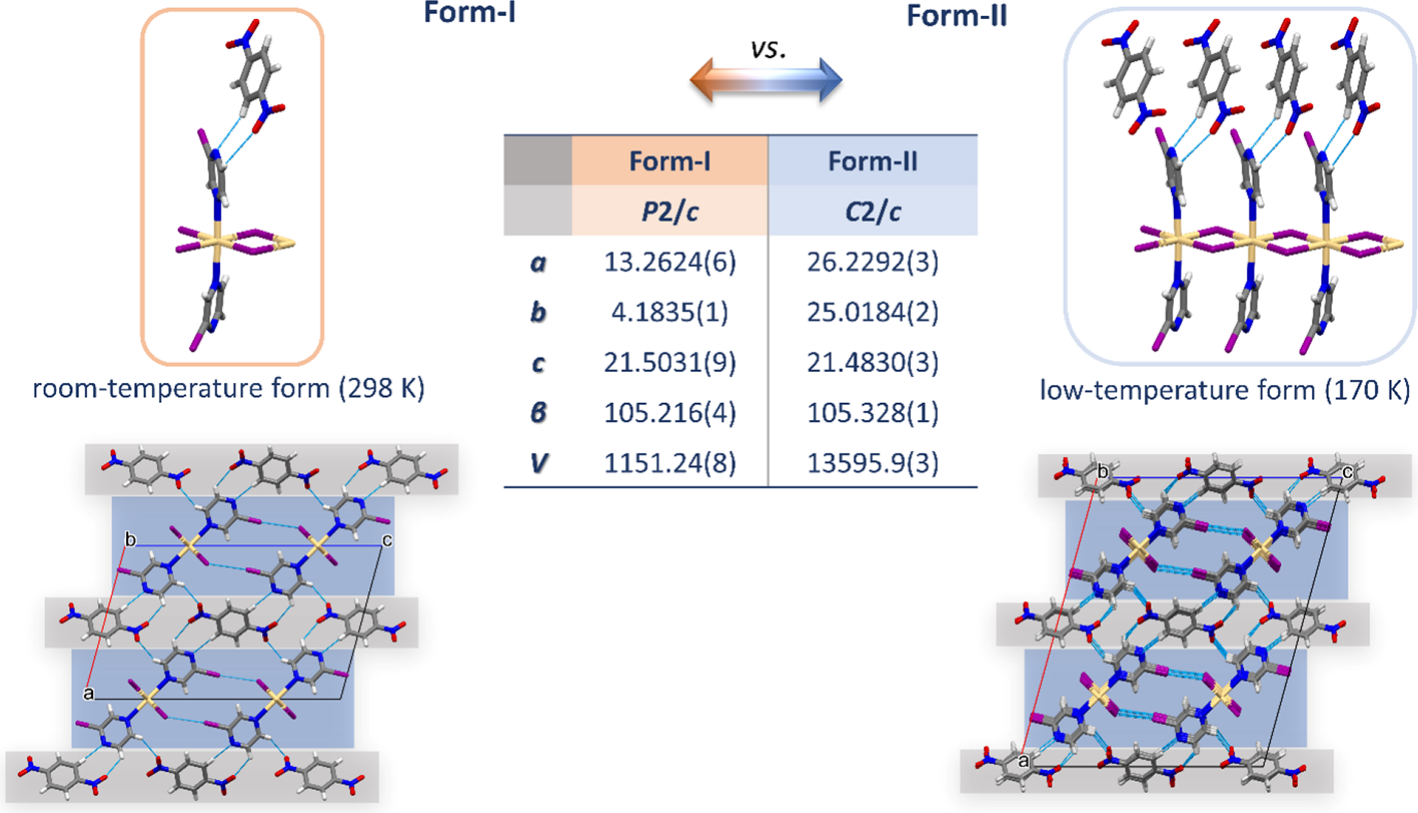
Room-temperature (Form-I; left) and low-temperature form (Form-II; right) of **1:B**.

In contrast to **1:A**, **1:B** at a low temperature presented a new polymorphic form (Form-II; LT). While Form-I (the room-temperature form, RT) contained all the features observed in **1:A**, Form-II displayed a lower symmetry of both polymeric building units and adjusted co-former molecules, which is impacting the overall corrugation of the structure (Figure 4). The outcome is a substantial enlargement of the polymeric unit used to describe the polymeric chain (tripling) as well as the unit cell (doubling of *a* and 6-fold enlargement of *b*). The supramolecular connectivity was preserved, and the same halogen and hydrogen bonds were observed in both forms, with only a small shortening (1–2%) of the donor–acceptor distances in Form-II (Figure 4 and Table S5). Each co-former (**B**) thus spans four polymeric units from two 2-D layers via two C_B_–H···N_CP_ and four C_CP_–H···O_B_ hydrogen bonds.

**Figure 5:**
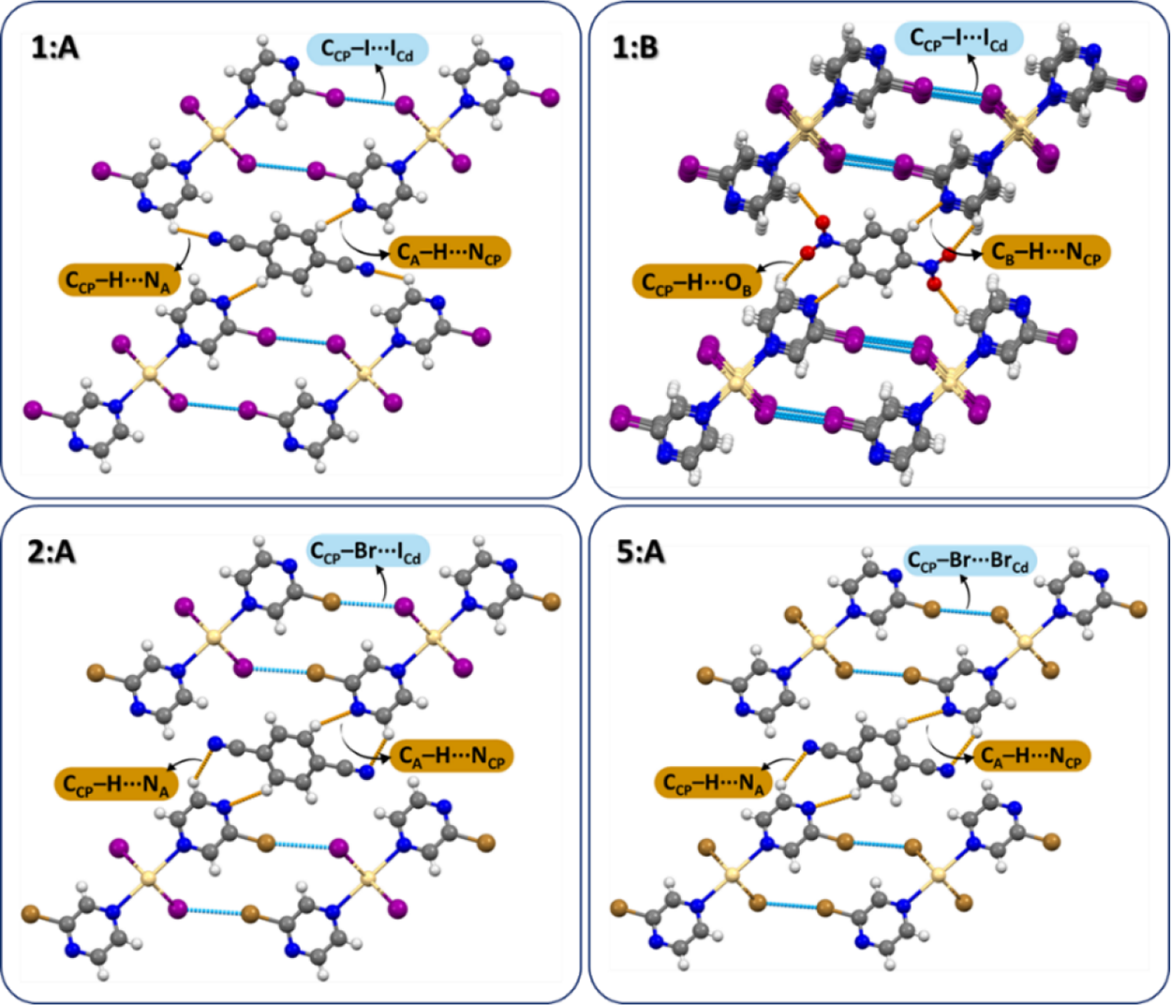
Crystal packing of **1:A**, **1:B**, **2:A**and **5:A**.

Two new materials, **2:A** and **5:A**, crystallize in monoclinic (*P*2/*c*) and triclinic (*P*1̅) space groups, respectively, but their structures did not reveal any surprises and showed the arrangement of the parent CP and co-former building units already observed in the structures of **1:A** (LT and RT) and **1:B** (RT). The links between the alternating CP–co-former layers are established through two C_A_–H···N_CP_ and two C_CP_–H···N_A_ hydrogen bonds, with a sole difference of both C–H···N hydrogen bonds being established between two polymeric units (instead of four as in **1:A** and **1:B**; Figure 5).

### Rationalization of the Synthetic Outcomes

Solvent-assisted grinding of seven co-formers with nine CPs yielded six co-crystals according to the PXRD screening (**1:A**, **1:B**, **2:A**, **3:A**, **5:A**, and **6:A**). Unfortunately, the attempts to obtain X-ray-quality single crystals from the resulting powder products gave only four co-crystalline materials suitable for structural characterization (**1:A**, **1:B**, **2:A**, and **5:A**). Moreover, **1:B** presented two polymorphic forms, room-temperature form, Form-I, and low-temperature form, Form-II, while the same was not observed for any of the co-crystals with co-former **A**, i.e., **1:A**, **2:A**, and **5:A** presented only one form.

Of the seven co-formers selected for the initial co-crystal screening (**A**, **B**, **C**, **F**, **G**, **H**, and **K**), four of them (**A**, **B**, **F**, and **G**) presented substantially larger positive MEP values (>159 kJ mol^–1^ e^–1^) than the rest (**C**, **H**, and **K**), and these four co-formers comprised two pairs of constitutional isomers (1,4- and 1,3-cyano isomers, **A** and **F**, and 1,4- and 1,3-nitro isomers, **B** and **G**). Interestingly, of the four isomers, only 1,4-isomers (**A** and **B**) yielded co-crystal forms, while 1,3-isomers (**F** and **G**), despite presenting very similar MEP values, were “inactive” in the context of the co-crystal formation. The results consequently implied that very specific steric requirements might be imposed on small organic co-formers for being brought into coexistence with rigid 1-D building units of CPs and delivering a stable co-crystal form.

Furthermore, co-former **A** contained a more positively charged (more “activated”) hydrogen atom (168 kJ mol^–1^ e^–1^) than **B** (159 kJ mol^–1^ e^–1^) and formed five co-crystals, while **B** yielded only one. This clearly demonstrates the importance of hydrogen-atom charge/activation for successful co-crystal formation. The remaining three co-formers with MEPs <160 kJ mol^–1^ e^–1^ (**C**, **H**, and **K**) did not produce any co-crystal, further indicating that only higher activated aromatic hydrogen atoms (i.e., relatively good hydrogen-bond donors) are capable of forming hydrogen bonds with the CP.

The co-formers that were not probed in the initial screening with nine CPs (**D**, **E**, **I**, **J**, **L**, and **M**) presented smaller MEP values at the hydrogen atoms and would therefore not be expected to compete successfully for the CPs’ acceptor sites. To test the validity of this assumption, we performed additional co-crystal screening experiments with the co-formers from *group I* that remained (**D** and **E**), and indeed, no co-crystals were obtained (Figures S9 and S10). The remaining co-formers from *groups III* and *IV* (**I**, **J**, **L**, and **M**) were exempt from further testing, since they were not equipped with additional anchoring sites (i.e., acceptor sites at electron-withdrawing moieties), which according to the results delivered in this study were a prerequisite for the co-crystal formation.

Regarding the CPs, out of nine CPs, five yielded co-crystals, three CdI_2_ (**1**–**3**), two CdBr_2_ (**5** and **6**), but none of the CdCl_2_-based CPs (**7**–**9**). Moreover, of the five CPs that successfully formed co-crystals, only **1** reacted with two co-formers (**A** and **B**), while the others produced co-crystals solely with one (**A**), indicating that even small differences in the power of hydrogen-bond donors/acceptors residing at the parent CPs (observed in slightly distinct MEP values at pyrazine H and N atoms; Figure 1) play a role in co-crystal delivery and are sufficient to impact the synthetic outcome.

Furthermore, **3** and **6**, even though they formed co-crystals with **A** (**3**:**A** and **6**:**A**), did not produce heteromeric materials upon dissolution in a variety of solvents. This demonstrated the pronounced sensitivity of **3:A** and **6:A** to changes in external conditions (primarily, polarity of solvents) and the presence of competing donor/acceptor sites.^31^ Moreover, the only co-crystal formed with **B** (**1:B**) yielded two polymorphic forms, room-temperature form (Form-I) and low-temperature form (Form-II), thus emphasizing the relevance of additional anchoring (i.e., C–H···O/N hydrogen bonds; four HBs in **1:B** vs two HBs in **1:A**, **2:A**, and **5:A**) in the stability of the co-crystal as well as flexibility of its supramolecular assembly to accommodate small-scale structural adjustments induced by temperature changes.

### Surface Site Interaction Point Pairing Energies

The observation of co-crystal formation between **A** and only five out of nine CPs was further rationalized based on pairing energies calculated via a simplified approach derived from Hunter’s work.^27,32^ In general, the likelihood of the co-crystal formation is evaluated on the surface site interaction point (SSIP) pairing energy difference (Δ*E*) between the co-crystal (*E*_cc_) and the two initial forms (coordination polymer, *E*_CP_, and co-former, *E*_cf_); the more negative the energy difference, the more probable co-crystal formation. The pairing energy for each form was calculated using the hydrogen-bond parameters α_i_ and β_i_, associated with the hydrogen-bond donor and acceptor sites, respectively, but only for the hydrogen bonds observed in the crystal structures of both pure forms (CPs and co-formers) and co-crystals formed (*E* = −Σα_i_ β_i_; for details, see the SI).

Since no surprises in the overall supramolecular networks were encountered, yet both the CPs (**1**–**9**) and co-crystals (**1:A**, **2:A**, and **5:A**) presented two groups with almost identical crystal structures, a comparison of the co-crystal formation energies was a relatively straightforward process. The SSIP pairing energies for both pure forms (*E*_CP_ and *E*_cf_) and potential co-crystals (**1:A**–**9:A**; *E*_cc_) were calculated, presuming that the same set of HBs would form in all co-crystals (Table 1), and the likelihood of the co-crystal formation was then rationalized within the framework of the energy differences (Δ*E*). The more negative energy difference (Δ*E*), the stronger the interactions between two co-crystal formers, thus favoring the co-crystal formation.

**Table 1:** Interaction Pairing Energies (kJ mol^–1^) for Existing and Potential Co-Crystals Formed between **1**–**9** and **A**

	*E*_cc_	*E*_CP_	*E*_cf_	Δ*E* = *E*_cc_ – (*E*_CP_ + *E*_cf_)
**1**	–32.48	–11.54	–20.52	–0.42
**2**	–33.18	–11.55	–20.52	–1.11
**3**^a^	–33.50	–11.34	–20.52	–1.64
**4**^b^	–32.60	–15.76	–20.52	3.68
**5**	–33.16	–15.72	–20.52	3.08
**6**^a^	–33.80	–15.48	–20.52	2.20
**7**^b^	–32.90	–18.18	–20.52	5.80
**8**^b^	–33.58	–18.89	–20.52	5.83
**9**^b^	–34.05	–19.20	–20.52	5.67

a Co-crystals formed but would not produce heteromeric co-crystals upon slow evaporation aimed at growing single crystals for structural characterization.

b Co-crystals not formed.

While only small differences in energies of co-crystals were observed (Δ*E*_cc_ < 2 kJ mol^–1^), the differences in energies of the parent CPs were more noticeable (Δ*E*_CP_ < 8 kJ mol^–1^) and followed the trend of the bridging halide acceptor power (Cl > Br > I) (the CdCl_2_ CPs (**7**–**9**) being the most stable while the CdI_2_ (**1**–**3**) being the least stable ones). The energies of the CPs reflected the likelihood of the formation of co-crystals themselves, suggesting that the co-crystals of the CdI_2_ CPs were the most likely to form, while the ones of CdCl_2_ were least likely to form co-crystals. Indeed, all three co-crystals of CdI_2_-based CPs, **1:A**–**3:A**, were formed via solvent-assisted grinding, only **3:A** did not yield single crystals upon dissolution in a variety of solvents (employed for gaining X-ray-suitable single crystals). Of the three co-crystals with CdBr_2_, **4:A**–**6:A**, two formed via grinding (**5:A** and **6:A**), but one (**6:A**) did not produce a heteromeric material upon dissolution but yielded rather two pure forms (**6** and **A**). The co-crystals with CdI_2_, **7:A**–**9:A**, were, according to the calculated SSIP pairing energy, the most disfavored ones, and they indeed did not form.

The SSIP calculated energies, despite relying on a simplified approach, revealed a satisfactory correlation with the experimental observations, thus indicating their practical further application for *a priori* narrowing down the co-crystal screening space.

## Conclusions

In this work, we have explored the ability of 1-D CPs to co-crystallize with small organic molecules, with the aim to extend a potential set of effective and reliable co-formers for modulating the mechanical properties of crystalline 1-D CPs. To streamline expensive and time-consuming experimental efforts, the MEP-based synthetic strategy was used to narrow the initial set of 13 co-formers with activated hydrogen-bond donors to only the most promising ones. This strategy was validated by the subsequent experimental co-crystal screen. Only co-formers having the most activated hydrogen atoms were incorporated into the CP’ crystal structure. Moreover, the accommodation of co-formers also proved very sensitive to steric influences, with only co-formers having a 1,4-relative arrangement of electron-withdrawing moieties (employed for the activation of hydrogen atoms) being successfully contained. All the structurally characterized co-crystals confirmed the existence of the intended hydrogen bonding between the CPs and co-formers, i.e., the C_cf_–H···N_CP_, critical for modulating mechanical performances of CPs. Furthermore, rationalization of the synthetic outcomes against the SSIP pairing energies was very useful for additionally reducing the co-crystal screening efforts. This theoretical tool, combined with the MEP-based synthetic strategy, constitutes a practical protocol for effectively narrowing the co-crystallization search space of CPs with small organic co-formers.

## Experimental Section

### Materials and Methods

Unless stated differently, all solvents and reagents were purchased from commercial suppliers and used without further purification. Starting 1-D CPs, [CdI_2_(I-pz)_2_]_*n*_ (**1**), [CdI_2_(Cl-pz)_2_]_*n*_ (**3**), [CdBr_2_(I-pz)_2_]_*n*_ (**4**), [CdBr_2_(Br-pz)_2_]_*n*_ (**5**), [CdBr_2_(Cl-pz)_2_]_*n*_ (**6**), [CdCl_2_(I-pz)_2_]_*n*_ (**7**), [CdCl_2_(Br-pz)_2_]_*n*_ (**8**), and [CdCl_2_(Cl-pz)_2_]_*n*_ (**9**), were prepared following literature procedures,^19,24^ synthesis of [CdI_2_(Br-pz)_2_]_*n*_ (**2**) was conducted by analogy with the former (see the SI), while synthesis of co-former, bis(diiodoethynyl)benzene (**H**), followed a two-step procedure described in detail in the SI.

### Mechanochemistry

Grinding was carried out in a Retsch MM200 ball mill. The standard reaction parameters were a milling time of 30 min with a frequency of 25 Hz, steel grinding balls with a diameter of 7 mm, and 10 mL stainless-steel jars.

### PXRD

PXRD experiments were performed on a Malvern Panalytical Aeris powder diffractometer (Cu *K*_α_ radiation, voltage 40 kV, and current 15.0 mA). Patterns were collected in the angle region between 5 and 40° (2θ) with a step size of 0.02°.

### Co-Crystal Screening

The initial co-crystal screening was carried out via solvent-assisted grinding using ethanol. Starting 1-D CPs (**1**–**9**) were combined with the co-formers (**A–C**, **F–G**, **H**, and **K**) in stoichiometric ratios and ground with 40 μL of ethanol (a total of 63 experiments). The ground mixtures were analyzed using PXRD to determine whether a co-crystal had formed or not. Successful co-crystal products were identified using new peaks (below 10° 2θ) in the PXRD patterns upon a comparison of PXRD patterns of a ground mixture with corresponding reactants, **1**–**9** and **A–C**, **F–G**, **H**, and **K**.

### TGA/DSC

Thermal analyses were performed for co-crystal products **2:A**, **3:A**, **5:A**, and **6:A**. Powder samples were heated from room temperature up to 600 °C in the nitrogen atmosphere (for details, see the SI).

### FT-IR

Infrared spectroscopy analyses were performed for all co-crystal products (**1:A**, **1:B**, **2:A**, **3:A**, **5:A**, and **6:A**) using the ATR technique. Data was collected in the wavelength range of 4000–400 cm^–1^ with a resolution of 4 cm^–1^ (details are provided in the SI).

### Growing Crystals

For each successful co-crystallization, the resulting solid was dissolved in a minimal volume of solvent (methanol, ethanol, acetone, and acetonitrile). A vial was then sealed using perforated parafilm and left undisturbed for slow evaporation to obtain crystals suitable for SCXRD. Colorless/yellowish crystals were harvested after a couple of days.

### Single-Crystal X-ray Crystallography

Suitable crystals for single-crystal X-ray experiments were isolated from the mother liquor and mounted in a random orientation on a glass fiber. Data collections were carried out on an XtaLAB Synergy-S Dualflex diffractometer with a PhotonJet (Mo) microfocus X-ray source and HyPix-6000HE hybrid photon counting (HPC) X-ray area detector and applying the CrysAlisPro Software system^33^ at 170(1) K. Data reduction, including absorption correction, was done by the CrysAlisPro program. The structures were solved by the SHELXT^34^ (**1:A**, **2:A**, **5:A**, **1:B**) and SHELXS^41^ (**2**) program. The coordinates and the anisotropic thermal parameters for all non-hydrogen atoms were refined by full-matrix least-squares methods based on *F*^2^ using the SHELXL program. The hydrogen atoms were generated geometrically using the riding model with the isotropic factor set at 1.2*U*_eq_ of the parent atom.

Graphical work has been performed by Mercury 4.3.1.^35^ The thermal ellipsoids were drawn at the 50% probability level. General and crystal data with the summary of intensity data collection and structure refinement for compounds **2**, **1:A**, **2:A**, **5:A**, and **1:B** are given in Table S1.

CCDC 2262418–2262422 contain the supplementary crystallographic data for this paper.

### Computational Details

Co-formers **A**–**M** and 1-D CPs **1**–**9** were optimized in CRYSTAL17^36^ with PBE^37^ functional and Grimme’s D3 correction for a better description of weak dispersive interactions.^38^ The revised triple-ζ basis set specifically adapted for periodic calculations, pob-TZVP-rev2, was used on all atoms.^39^ Full optimization of atom coordinates and cell parameters was performed on the starting geometries. Tighter convergence on total energy (10^–7^) and increased truncation criteria for the calculation of Coulombs and exchange integrals (8 8 8 8 16) were set for SCF calculations. Cube files were generated with CRYSTAL17 and visualized in GaussView 6.^40^ The ESP values were plotted onto the total electron density isosurface (isovalue of 0.002 a.u.). The minimum and maximum values were read directly from the plot.

### Using the Calculated Hydrogen-Bond Energies for Rationalization of Structural Outcomes

The SSIP pairing energy was calculated based on the approach using MEP values associated with the sites in the hydrogen-bond interaction. The parameters α_i_ and β_i_, typically associated with the hydrogen-bond donor and acceptor sites, were calculated using maxima and minima on the MEP surfaces, respectively, and the energy of interactions was derived by summing their products (−α_i_ β_i_) for each hydrogen-bond interaction realized in the crystal structure:
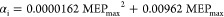

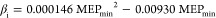

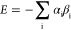


The probability of co-crystal formation was estimated on the difference in the SSIP pairing energy (Δ*E*) between the co-crystal (*E*_cc_) and the two pure forms (the parent coordination polymer, *E*_CP_, and co-former, *E*_cf_) in a 1:1 stoichiometric ratio:

Δ*E* = *E*_cc_ – (*E*_CP_ + *E*_cf_)

A positive Δ*E* value means that the two initial pure forms are more stable than the intended co-crystal (i.e., the co-crystals do not form), while a negative Δ*E* value indicates that the co-crystal itself is more stable than the two forms (i.e., the co-crystal forms). Furthermore, the more negative the Δ*E*, the stronger the pairing energy between the two forms.

## Supporting Information Available

Synthetic procedures for the preparation of compounds **2**, **1:A**, **1:B**, **2:A**, **3:A**, **5:A**, and **6:A**, PXRD, IR and TGA/DSC results, SCXRD results, and computational studies (PDF)
