# Supplementary material for: Exploring the Co-Crystallization Landscape of One-Dimensional Coordination Polymers Using a Molecular Electrostatic Potential-Driven Approach
Source: Cryst Growth Des. 2023 Sep 1;23(10):7198–206. doi: 10.1021/acs.cgd.3c00615 (PMC11010263; doi:10.1021/acs.cgd.3c00615)
Supplement: Supplementary file 1 — cg3c00615_si_001.pdf [file cg3c00615_si_001.pdf]

Exploring the co-crystallization landscape of 1-D coordination polymers using an MEP-driven approach

Ozana Mišura,[a] Ivan Kodrin,[a] Mladen Borovina,[a] Mateja Pisačić,[a] Viraj De Silva,[b] Christer B. Aakeröy,[b] and Marijana Đaković[a*]

[a]*Department of Chemistry, Faculty of Science, University of Zagreb, Zagreb, Croatia*

[b]*Department of Chemistry, Kansas State University, Manhattan, KS 66506 USA*

Supplementary information

Table of Contents

1. Synthesis 3

2. Co-crystal screening and growing crystals 5

3. Powder X-ray crystallography 7

4. Single-crystal X-ray crystallography 14

5. Computational studies / Molecular electrostatic potential maps 22

6. Thermogravimetric analysis (TGA) and Differential Scanning Calorimetry (DSC) 25

7. Infrared Spectroscopy (FT-IR) 27

References 29

1. Synthesis

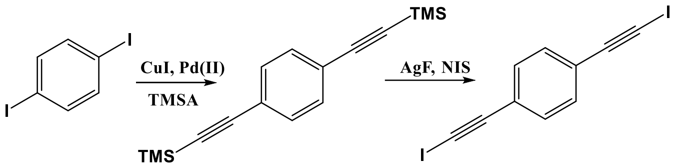

Scheme S1. Synthesis of co-crystallizing compound 1,4-bis(iodoethynyl)benzene (**H**).

Synthesis of 1,4-bis((trimethylsilyl)ethynyl)benzene

1,4-Diiodobenzene (0.02 mol, 6.598 g) was dissolved in a mixture of triethylamine (50 mL) and tetrahydrofuran (50 mL) and purged with nitrogen gas for 10 minutes followed by the addition of CuI (0.002 mol, 0.380 g), bis(triphenylphosphine)palladium(II) chloride (0.001 mol, 0.701 g) and trimethylsilylacetylene (0.08 mol, 11.52 mL). The mixture was stirred overnight under nitrogen at 75 °C. Upon completion the reaction mixture was cooled to room temperature and the solvent was evaporated using a rotary evaporator. The crude was purified by column chromatography. Yield: 75%; MP: 115–122 °C; ^{1}H NMR (400 MHz, Chloroform-*d*) δ 7.38 (s, 4H), 0.24 (s, 18H); ^{13}C NMR (101 MHz, Chloroform-*d*) δ 131.89, 123.29, 104.70, 96.44, 0.05.

Synthesis of 1,4-bis(iodoethynyl)benzene

1,4-bis((trimethylsilyl)ethynyl)benzene (0.0074 mol, 2.001 g) was dissolved in acetonitrile/toluene (50:50 mL) and was purged with N_2 for 10 min. This was covered with aluminum foil and AgF (0.0222 mol, 2.799 g) and N-Iodosuccinimide (0.0222 mol, 5.439 g) were added. This was stirred for 24 hours at room temperature and upon completion the reaction mixture was passed through a silica plug and the filtrate was concentrated to obtain the crude. The crude was redissolved in diethyl ether and washed with saturated sodium thiosulfate and water and dried over $MgSO_4$ and the organic layer was concentrated. The crude was passed through a flash column with hexane to obtain the pure product. Yield: 40%; Decomp: 185–195 °C; ^{1}H NMR (400 MHz, Chloroform-*d*) δ 7.35 (s, 4H); ^{13}C NMR (101 MHz, Chloroform-*d*) δ 132.26, 123.84, 93.70, 9.23.

Preparation of $[CdI_2(Br\text{-}pz)_2]_n$ (2)

Cadmium(II) iodide (CdI_2, 1 eq.) was dissolved in water (5 mL) and bromopyrazine (Br-pz, 2 eq.) was dissolved in ethanol (96%, 5 mL). The resulting colourless solutions are mixed together and stirred with a magnetic stirrer. After a few minutes a white precipitate was formed, and stirring was continued for additional 20 minutes. The product was filtered off, washed with small amounts of cold water and left to dry at atmospheric conditions.

CHN analyses were performed with a Perkin-Elmer 2400 Series II CHNS analyzer in the Analytical Services Laboratories of the Ruđer Bošković Institute, Zagreb, Croatia.

Used: CdI_2 (0.10 g, 0.27 mmol), Br-pz (105 μL, ρ = 1.73 g cm^{-3}; 1.14 mmol). Microanalysis. Calc. for $C_8H_6CdBr_2I_2N_4$ (M_r = 684.19): C, 14.04; H, 0.88; N, 8.19 % Found: C, 13.88; H, 0.70; N, 8.0 %.

The crystallization of the $[CdI_2(Br\text{-}pz)_2]_n$ (**2**) was carried out, to acquire the crystal structure, as previously reported crystal structure in the *Cambridge Structural Database*;[1] *refcode*: **YEFMEE**, is another polymorph with different unit cell parameters.

Growing crystals of $[CdI_2(Br\text{-}pz)_2]_n$ (2)

Cadmium(II) iodide (1 eq.) was dissolved in water and bromopyrazine (2 eq.) in ethanol (w = 96%). The resulting water solution of the cadmium(II) iodide was added to a test tube and first carefully layered with 1 mL of ethanol (w = 96%), then the ethanol solution of the ligand was carefully added to the ethanol layer. The test tube was sealed using parafilm and left undisturbed with a few holes punctured in the parafilm. Colorless X-ray quality needle-like crystals were harvested after a couple of weeks.

2. Co-crystal screening and growing crystals

The reaction mixture of 1-D coordination polymer (**1**–**9**) (1 eq.) and organic co-former (**A**–**C**, **F**–**G**, **H** and **K**) (1 eq.) was placed in a 10 mL stainless steel jar with 40 µL of ethanol and 2 stainless steel balls (7 mm in diameter) and ground for 30 minutes at 25 Hz frequency. Sixty-three screening reactions in total were performed. Out of all performed grinding reactions, six co-crystalline products were successfully obtained according to the PXRD analysis; **1:A**, **2:A**, **3:A**, **5:A**, **6:A**, and **1:B**.

To ensure pure product delivery, several conditions were varied: the grinding-assisted solvent (ethanol, methanol, acetone), washing of the ground product with small amounts of cold solvent, the length of the grinding (30, 40, 60 or 90 minutes), and the excess amount of co-former (10%, 20%). For **1:A** and **2:A**, extended grinding time (60 min); for **5:A**, synthesis with the excess of co-former (1 eq./1.1 eq.), followed by washing the resulting mixture with cold ethanol; for **1:B**, re-crystallizing the primary-conditioned grinding mixture from methanol, resulted in the pure product. In the case of **3:A** and **6:A**, isolation of the pure product was not successful.

Co-crystal synthesis

1:A. Used: $[CdI_2(I\text{-}pz)_2]_n$ (100.3 mg; 0.129 mmol) and 1,4-dicyanobenzene (17.1 mg; 0.133 mmol). Conditions: 40 µL of ethanol, 60 minutes, no washing of the resulting mixture.

2:A. Used: $[CdI_2(Br\text{-}pz)_2]_n$ (99.5 mg; 0.145 mmol) and 1,4-dicyanobenzene (18.7 mg; 0.146 mmol). Conditions: 40 µL of ethanol, 60 minutes, no washing of the resulting mixture.

5:A. Used: $[CdBr_2(Br\text{-}pz)_2]_n$ (99.1 mg; 0.168 mmol) and 1,4-dicyanobenzene (21.8 mg; 0.170 mmol). Conditions: co-former added with 10% excess, 40 µL of ethanol, 60 minutes, washing of the resulting mixture with cold ethanol.

1:B. Used: $[CdI_2(I\text{-}pz)_2]_n$ (99.7 mg; 0.128 mmol) and 1,4-dinitrobenzene (21.4 mg; 0.127 mmol). Conditions: 40 µL of ethanol, 60 minutes, no washing of the resulting mixture.

3:A. Used: $[CdI_2(Cl\text{-}pz)_2]_n$ (50.1 mg; 0.084 mmol) and 1,4-dicyanobenzene (10.9 mg; 0.085 mmol). Conditions: 40 µL of ethanol, 30 minutes, no washing of the resulting mixture.

6:A. Used: $[CdBr_2(Cl\text{-}pz)_2]_n$ (50.4 mg; 0.101 mmol) and 1,4-dicyanobenzene (13.0 mg; 0.101 mmol). Conditions: 40 µL of ethanol, 30 minutes, no washing of the resulting mixture.

General procedure for growing crystals by re-crystallization

The resulting mechanochemical product (**1:A**, **2:A**, **3:A**, **5:A**, **6:A**, and **1:B**) was placed in a test tube and dissolved in a variety of solvents and solvent mixtures: methanol, ethanol, acetonitrile, acetone, propan-1-ol, *iso*propan-1-ol, ethanol/water (1:1), acetone/water (4:1), acetone/benzene (1:1), acetone/toluene (1:1), acetone/cyclohexane (1:1) and acetonitrile/acetone (1:1). The test tube with prepared solution was sealed with perforated parafilm and left undisturbed, whereas additional holes in parafilm were made after several hours. After a couple of days, crystals were observed in several solvents (methanol, acetone and acetonitrile). Four crystalline compounds (**1:A**, **2:A**, **5:A** and **1:B**) yielded X-ray-quality single crystals.

X-ray quality crystals were isolated from:
1:A: methanol and acetone; **2:A**: acetonitrile; **5:A**: acetone; **1:B**: methanol and acetonitrile.

Re-crystallizations of **3:A** and **6:A** resulted in either single crystals of poor quality (not appropriate for the SCXRD experiment) or yielded mixtures of starting components (coordination polymer, **3** or **6**, and 1,4-dicyanobenzene, **A**).

3. Powder X-ray crystallography

Polycrystalline samples were finely ground and placed on a silicon plate for powder X-ray diffraction (PXRD) experiments which were performed at room temperature on a Malvern Panalytical Aeris powder diffractometer in the Bragg-Brentano geometry with PIXcel^{1D} detector under applied voltage of 40 kV and current of 15.0 mA. The radiation used was CuK_{α} (λ = 1,5406 Å), and all patterns were collected at room temperature, from 5° to 40° (2θ) and with a step size of 0.02°. The PXRD data were collected for bulk samples.

For **2**, the PXRD pattern of the bulk sample was overlayed with the pattern calculated from the crystal structure (Figure S1).

To identify the successful co-crystal formation, PXRD patterns of respective 1-D coordination polymer and organic co-former were compared with the PXRD pattern collected for bulk products. For successful co-crystal formations, the overlays are presented in Figures S2–S7.

For co-crystals, for which the SCXRD structures were determined, the PXRD patterns of starting compounds (coordination polymer and co-former) and bulk samples were also compared with the patterns calculated from the crystal structure (Figures S2–S4, S6).

The collected powder patterns were analyzed and evaluated using Data Viewer software (version 1.9a).[2]

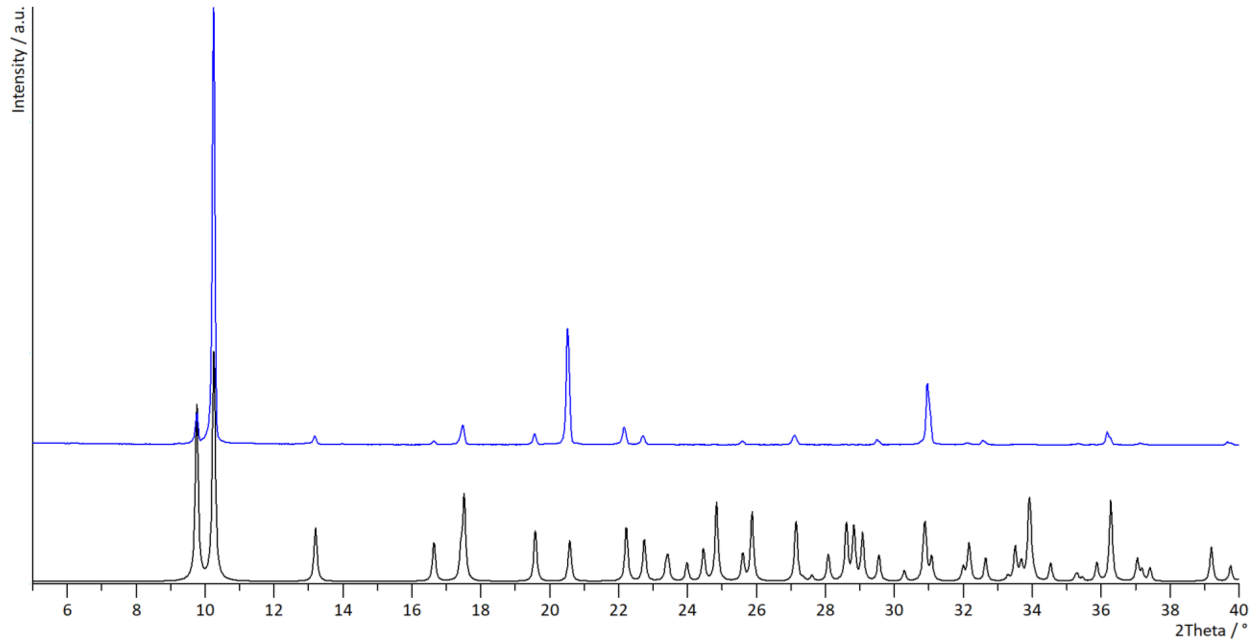

Figure S1. Overlay of PXRD traces $[CdI_2(Br\text{-}pz)_2]_n$ (**2**) calculated (**black**) and experimental (**blue**).

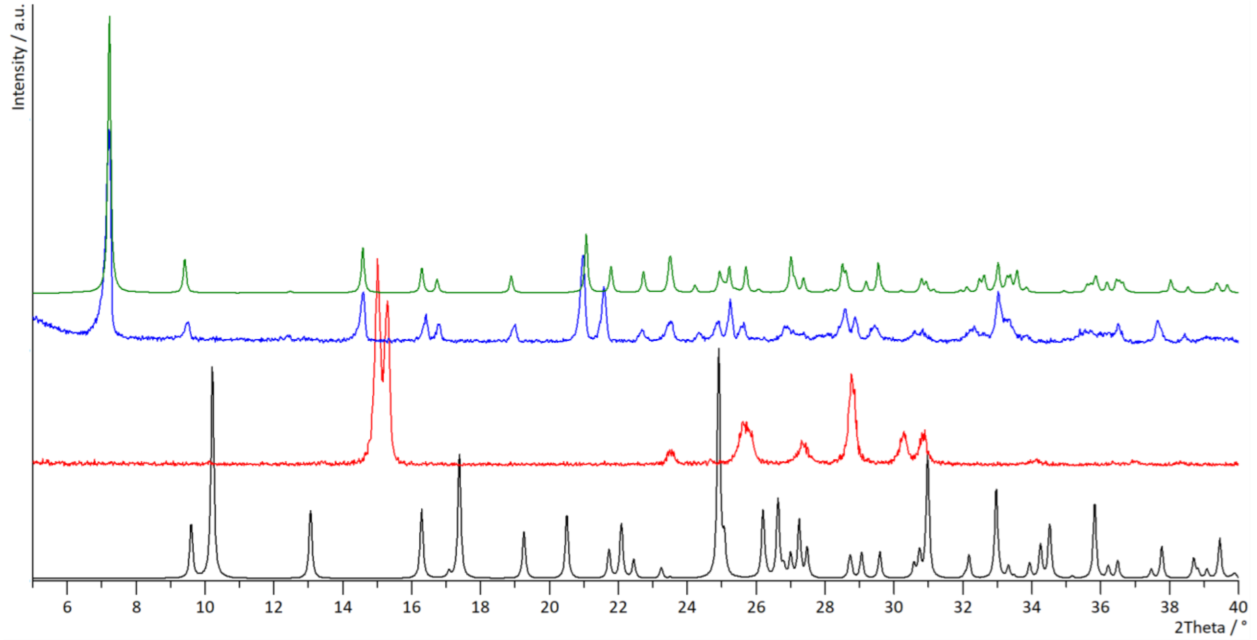

Figure S2. Overlay of PXRD traces of starting compounds, $[CdI_2(I\text{-}pz)_2]_n$ (**1, black;** *refcode:* **YIKJEK**) and 1,4-dicyanobenzene (**A, red**), and the resulting co-crystal **1:A** (bulk*,* **blue***,* and calculated*,* **green**).

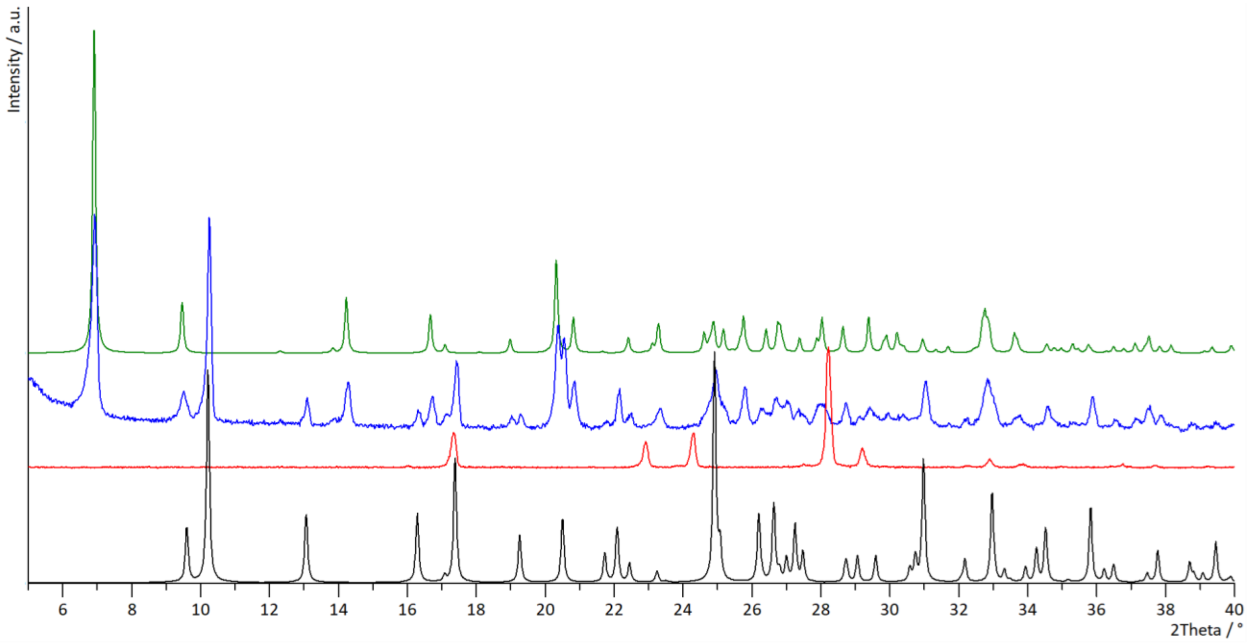

Figure S3. Overlay of PXRD traces of starting compounds, $[CdI_2(I\text{-}pz)_2]_n$ (**1, black;** *refcode:* **YIKJEK**) and 1,4-dinitrobenzene (**B, red**), and the resulting co-crystal **1:B** (bulk, **blue**, and calculated Form-I, **green**).

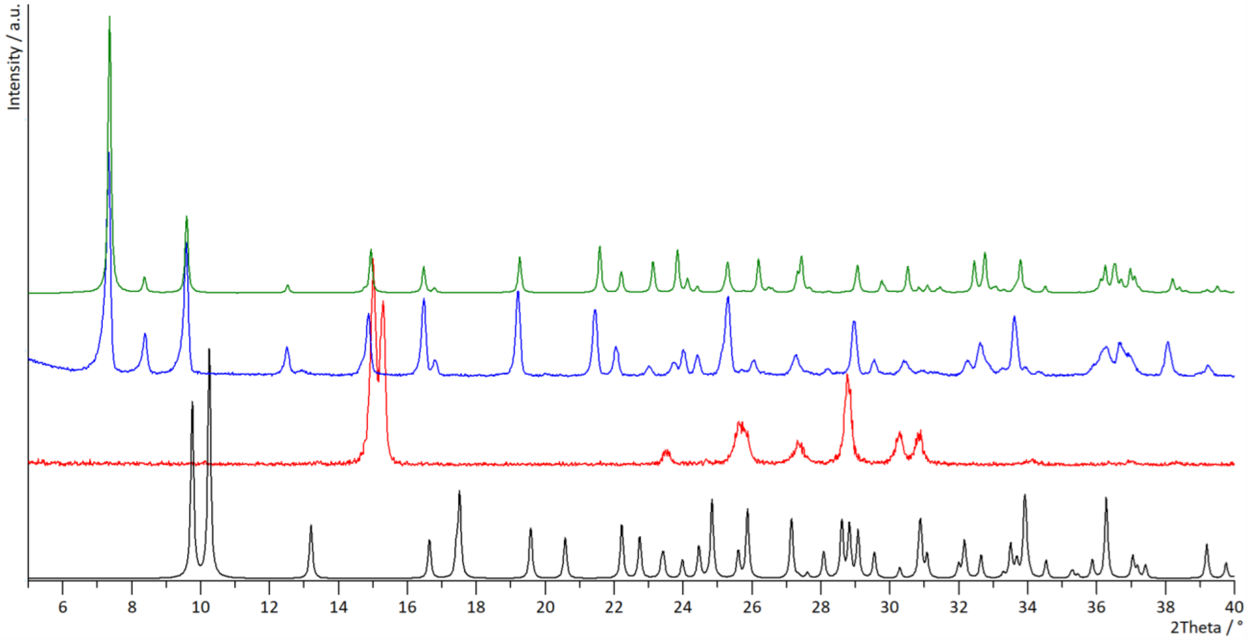

Figure S4. Overlay of PXRD traces of starting compounds, $[CdI_2(Br\text{-}pz)_2]_n$ (**2, black**) and 1,4-dicyanobenzene (**A, red**), and the resulting co-crystal **2:A** (bulk, **blue**, and calculated, **green**).

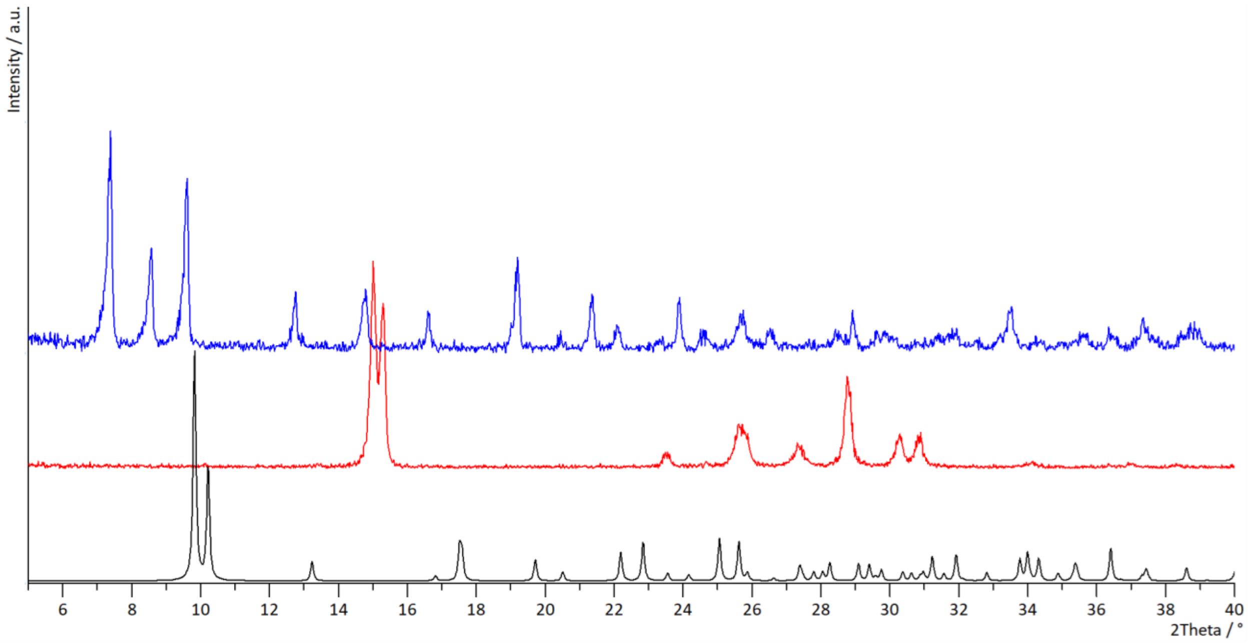

Figure S5. Overlay of PXRD traces of starting compounds, $[CdI_2(Cl\text{-}pz)_2]_n$ (**3, black;** *refcode:* **QAWGUT**) and 1,4-dicyanobenzene (**A, red**), and the resulting co-crystal **3:A** (**blue**).

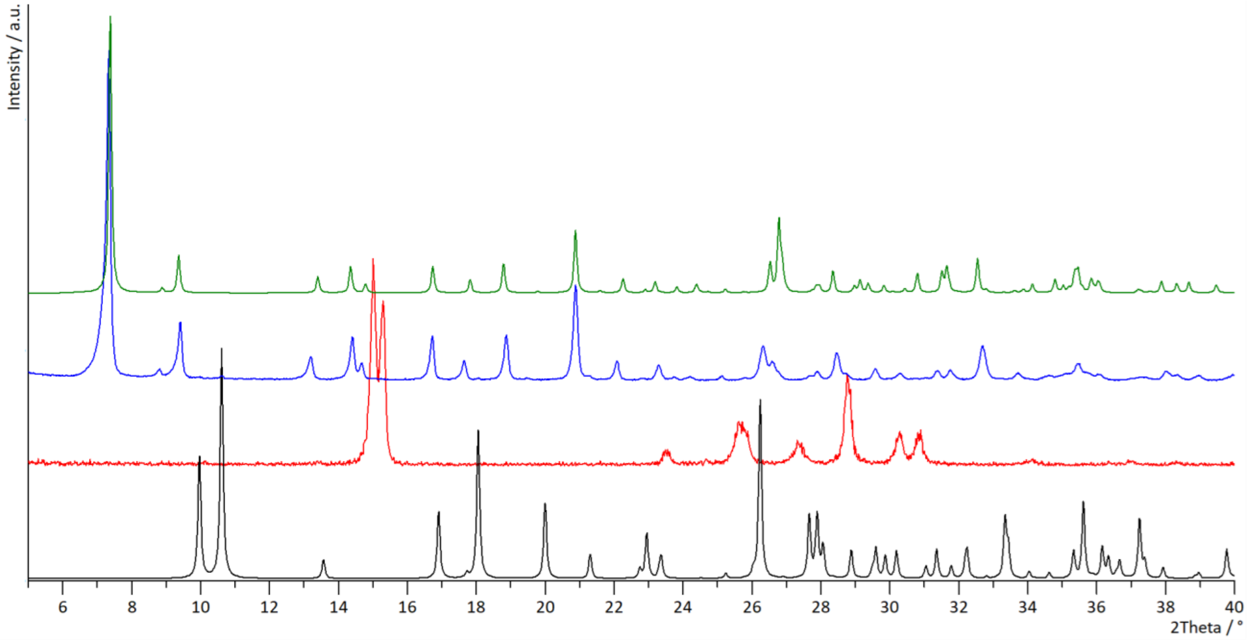

Figure S6. Overlay of PXRD traces of starting compounds, $[CdBr_2(Br\text{-}pz)_2]_n$ (**5, black;** *refcode:* **YIKJOU**) and 1,4-dicyanobenzene (**A, red**), and the resulting co-crystal **5:A** (bulk, **blue**, and calculated, **green**).

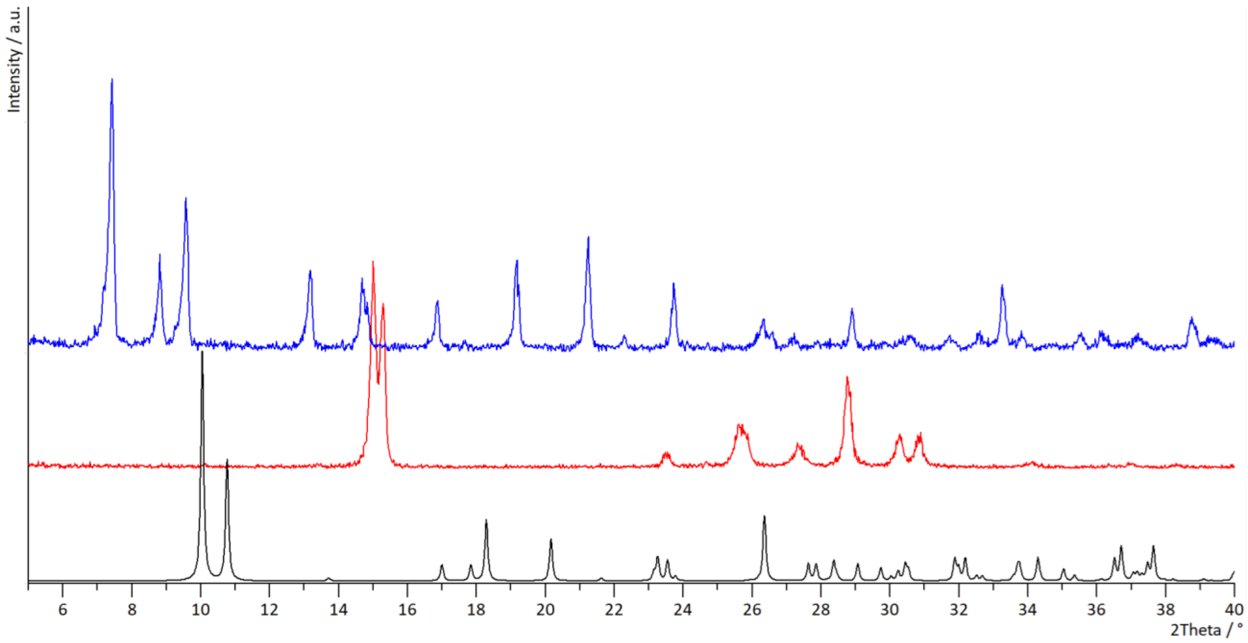

Figure S7. Overlay of PXRD traces of starting compounds, $[CdBr_2(Cl\text{-}pz)_2]_n$ (**6, black;** *refcode:* **QAWGON**) and 1,4-dicyanobenzene (**A, red**), and the resulting co-crystal **6:A** (**blue**).

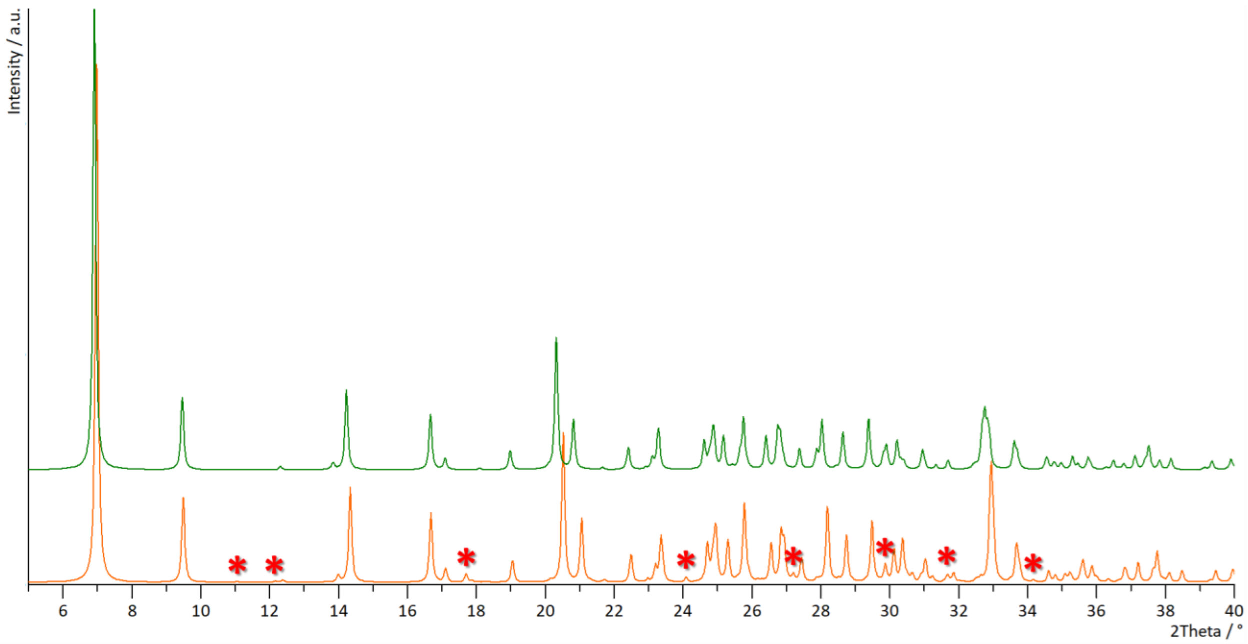

Figure S8. Comparison of PXRD patterns calculated from the crystal structures of **1:B** at low temperature (170 K, **orange**; Form-II) and room temperature (295 K, **green**; Form-I).

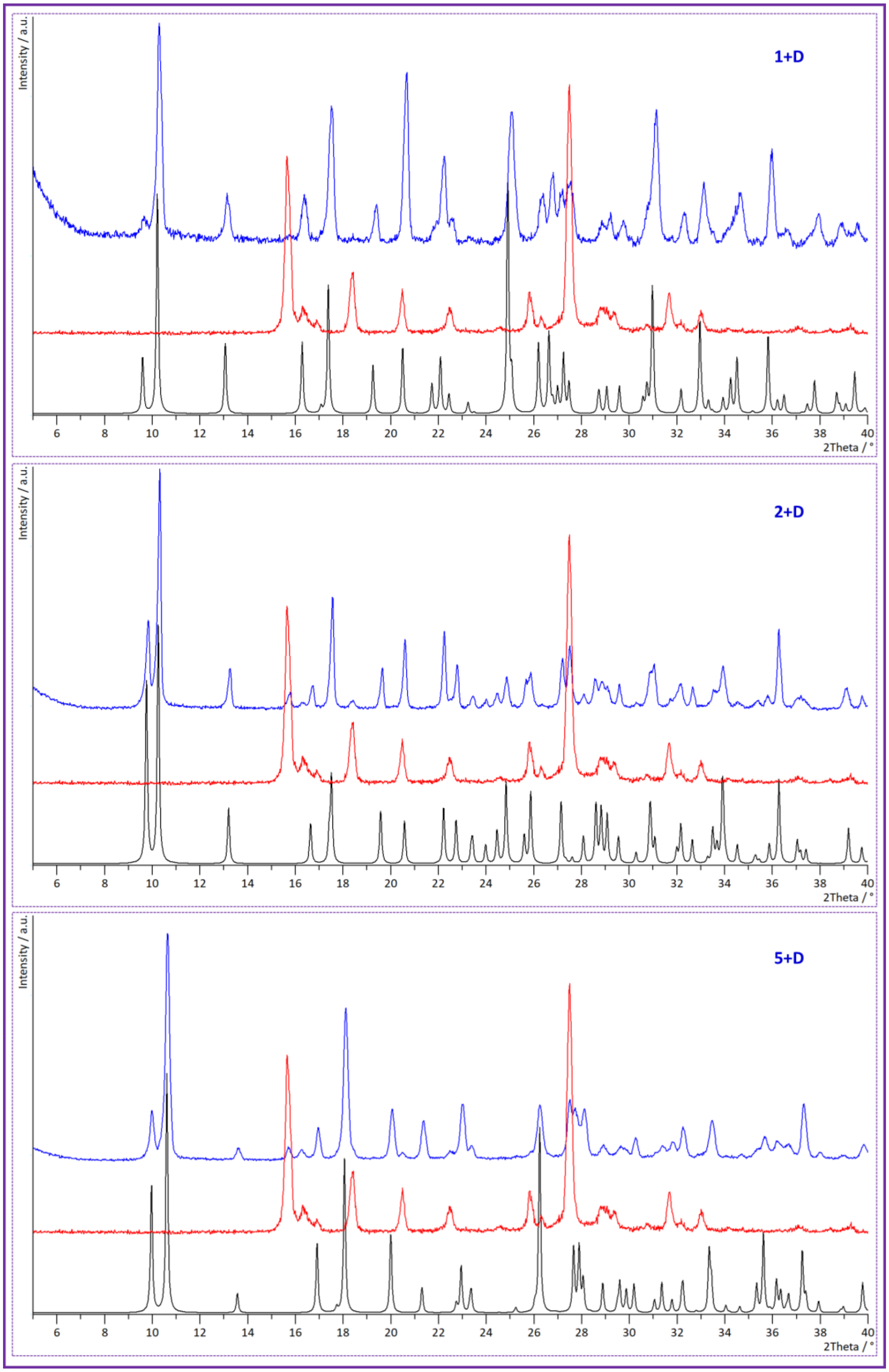

Figure S9. Overlays of PXRD patterns for resulting grinding products of **1** and **D** (**1+D**, top), **2** and **D** (**2+D**, middle) and **5** and **D** (**5+D**, bottom) with corresponding CP (**black**) and co-former **D** (**red**).

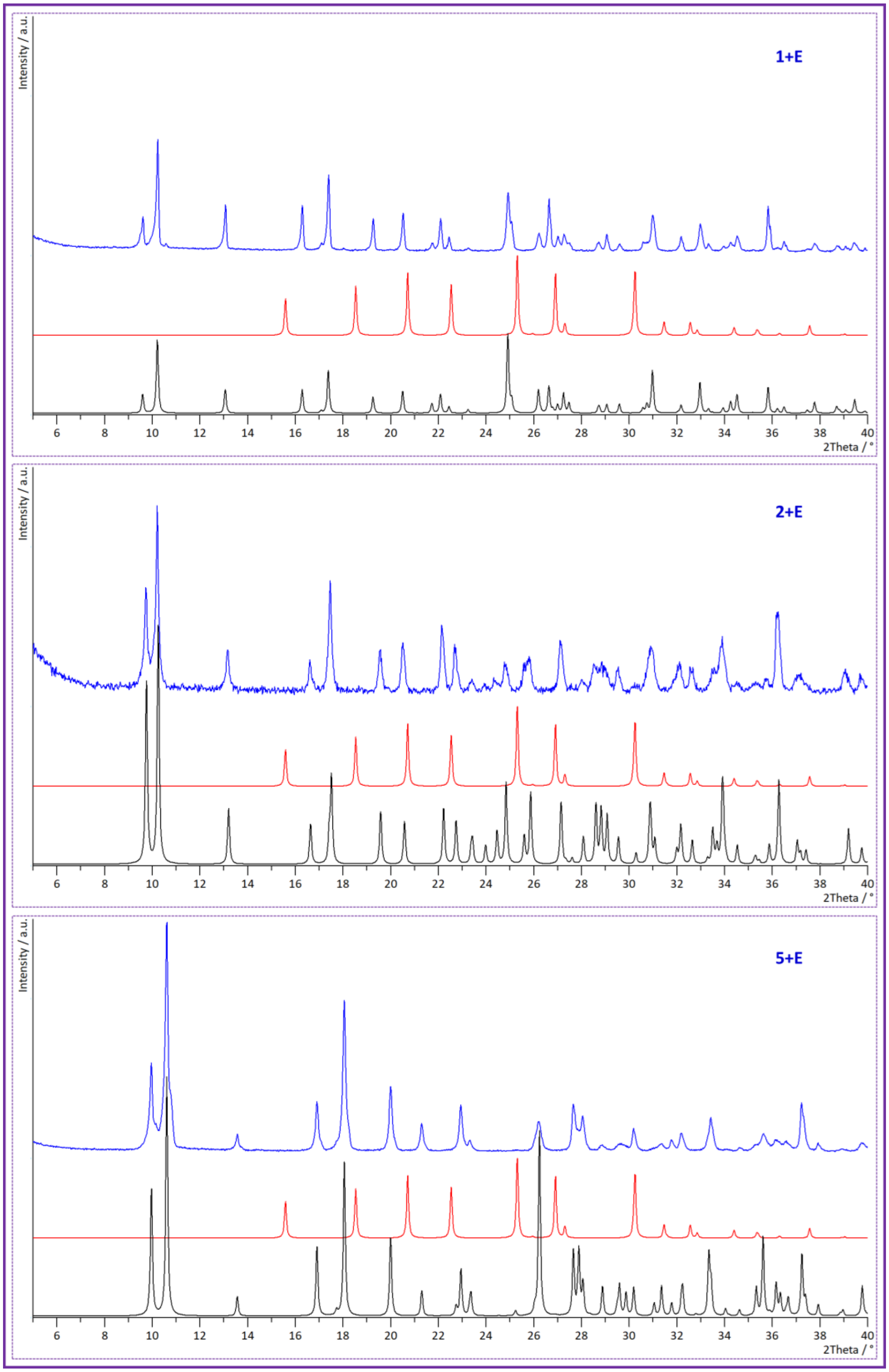

Figure S10. Overlays of PXRD patterns for resulting grinding products of **1** and **E** (**1+E**, top), **2** and **E** (**2+E**, middle) and **5** and **E** (**5+E**, bottom) with corresponding CP (**black**) and co-former **E** (**red;** *refcode*: **FACGEV**).

4. Single-crystal X-ray crystallography

Crystals of **2**, **1:A**, **2:A**, **5:A** and **1:B** suitable for single-crystal X-ray experiments were isolated from the mother liquor and mounted in a random orientation on a glass fibre. Data collections for **1:A**, **2:A**, **5:A** and **1:B** were carried out on the XtaLAB Synergy-S Dualflex diffractometer with PhotonJet (Mo) microfocus X-ray source and HyPix-6000HE hybrid photon counting (HPC) X-ray area detector, and applying the CrysAlisPro Software system[3] at 170(1) K, while data collections for **2** were carried out on the Oxford Diffraction Xcalibur four-circle kappa geometry single-crystal diffractometer with Sapphire 3 CCD detector, using a graphite monochromated MoK_{α} (λ = 0.71073 Å) radiation at 200(2) K, and applying the CrysAlisPro Software system.[1]

Data reduction, including absorption correction, was done by the CrysAlisPro program. The structures of **1:A**, **2:A**, **5:A** and **1:B** were solved by dual-space methods using the SHELXT[4] program, and the structure of **2** by direct methods using the SHELXS[5] program. The coordinates and the anisotropic thermal parameters for all non-hydrogen atoms were refined by full-matrix least-squares methods based on F^2 using the SHELXL[6] program. The hydrogen atoms were generated geometrically using the riding model with the isotropic factor set at 1.2U_{eq} of the carbon atom to which they are attached.

Space groups of structures **2, 1:A, 2:A** and **5:A** were determined unambiguously and no additional difficulties have arisen during their solution and refinement process. Correct space group determination was not as straightforward for **1:B** and Checkcif gave pseudosymmetry alerts. While compound **1:B** crystallizes in the *P*2/c space group at room temperature with cell parameters of *a* = 13.2476(7) Å, *b* = 4.1813(2) Å, *c* = 21.4857(11) Å, β = 105.152(4)°, at 170 K the same space group accounts for only 57% of reflections and clear signs of a supercell structure arise. It is possible to solve and refine the structure in the room temperature space group (*P*2/c) with a reasonably low *R* factor value (*R* = 2.46%), however, the displacement ellipsoids in the aromatic rings are unusually large and elongated, and a large residual electron density hole of –2.4 eÅ^3 appears around the Cd and I atoms. CrysAlisPro automatically detects *C*2/*c* as the space group with cell parameters of *a* = 26.2292(3) Å, *b* = 25.0184(2) Å, *c* = 21.4830(3) Å, β = 105.328(1)° which accounts for 97% of the reflections. When the structure is solved and refined in the latter space group (*C*2/*c*), the *R* value slightly rises (*R* = 2.81%), however, the residual electron density

is much smoother and featureless, and reasonable displacement ellipsoids are obtained. Attempts were also made to solve the structure in the $P2/c$ and $P\bar{1}$ space groups resulting in structures of inferior quality (regarding both R factors and displacement ellipsoids) than the one obtained in $C2/c$ and in both cases, the Addsym[7] algorithm in Platon[8] suggested the $C2/c$ space group. Systematic absences were also investigated in CrysAlisPro and they correspond with the $C2/c$ space group.

Graphical work has been performed by Mercury 4.3.1,[9] and the thermal ellipsoids were drawn at the 50% probability level. General and crystal data with the summary of intensity data collection and structure refinement for **2**, **1:A**, **2:A**, **5:A**, and **1:B** are given in **Table S1**.

CCDC 2262418–2262422 contain the supplementary crystallographic data for this paper.

Table S1. Crystal data and details of the structure determination for **2**, **1:A**, **2:A**, **5:A** and **1:B**.

**Compound**	**2**	**1:A**	**2:A**	**5:A**	**1:B**
Formula	$C_8H_6CdI_2Br_2N_4$	$C_{16}H_{10}CdI_4N_6$	$C_{16}H_{10}Br_2CdI_2N_6$	$C_{16}H_{10}Br_4CdN_6$	$C_{42}H_{30}Cd_3I_{12}N_{18}O_{12}$
M_r	684.19	906.30	812.32	718.34	2838.84
Color and habit	colorless, block	colorless, needle	colorless, needle	colorless, needle	yellowish, irregular
Crystal system, space group	Monoclinic, $P2_1/c$ (No. 14)	Monoclinic, $P2/c$ (No. 13)	Monoclinic, $P2/c$ (No. 13)	Triclinic, P-1 (No. 2)	Monoclinic, $C2/c$ (No. 15)
Crystal dimensions (mm^3)	0.07 x 0.15 x 0.54	0.02 x 0.03 x 0.24	0.02 x 0.03 x 0.3	0.03 x 0.04 x 0.61	0.04 x 0.11 x 0.38
a (Å)	4.1280(1)	12.7238(3)	12.4334(4)	3.8838(1)	26.2292(3)
b (Å)	10.6535(2)	4.1292(1)	4.0619(1)	10.6175(2)	25.0184(2)
c (Å)	17.2882(5)	22.0294(5)	21.8702(6)	12.7888(2)	21.4830(3)
α (Å)	90	90	90	69.538(2)	90
β (Å)	93.306(2)	106.033(2)	105.171(3)	87.336(1)	105.328(1)
γ (Å)	90	90	90	88.340(1)	90
V (Å^3)	759.03(3)	1112.39(5)	1066.02(5)	493.51(2)	13595.9(3)
Z	2	2	2	1	8
ρ_{calc} (g cm^{-3})	2.994	2.706	2.531	2.417	2.774
μ (Mo-K_α) (mm^{-1})	10.754	6.546	7.682	9.217	6.446
F(000)	612	816	744	336	10272
T temperature (K)	200(2)	170(1)	170(1)	170(1)	170(1)
λ radiation wavelength (Å)	Mo K_α	Mo K_α	Mo K_α	Mo K_α	Mo K_α
θ range for data collection (˚)	$3.04 \leq \theta \leq 31.99$	$2.87 \leq \theta \leq 30.99$	$2.89 \leq \theta \leq 30.99$	$2.05 \leq \theta \leq 30.99$	$1.90 \leq \theta \leq 31.00$
h, k, l range	$-4 \leq h \leq 5$<br>$-15 \leq k \leq 12$<br>$-24 \leq l \leq 25$	$-18 \leq h \leq 17$<br>$-5 \leq k \leq 5$<br>$-31 \leq l \leq 30$	$-18 \leq h \leq 18$<br>$-5 \leq k \leq 5$<br>$-31 \leq l \leq 31$	$-5 \leq h \leq 5$<br>$-15 \leq k \leq 15$<br>$-18 \leq l \leq 18$	$-38 \leq h \leq 38$<br>$-36 \leq k \leq 35$<br>$-31 \leq l \leq 31$
Scan type	ω	ω	ω	ω	ω
No. measured reflections	8364	21671	30087	31567	425475
No. independent reflections (R_{int})	2513	3513	3386	3162	21657
No. observed reflections, $I \geq 2\sigma(I)$	2091	3331	2830	2794	13383
No. refined parameters	79	123	123	124	784
R, wR [$I \geq 2\sigma(I)$]	0.0256, 0.0482	0.0145, 0.0365	0.0255, 0.0524	0.0248, 0.0594	0.0281, 0.0636
R, wR [all data]	0.0358, 0.0521	0.0159, 0.0369	0.0351, 0.0547	0.0303, 0.0609	0.0537, 0.0718
Goodness of fit on F^2, S	1.038	1.074	1.044	1.065	1.016
Max., min. electron density (e Å^{-3})	−0.828, 0.914	−0.521, 0.785	−0.511, 1.001	−0.726, 0.864	−0.637, 0.956
CCDC number	2262420	2262418	2262421	2262422	2262419

Table S2. Selected bond distances (Å) and angles (˚) for **1:A**, **2:A** and **2**.

	**1:A**	**2:A**		**2**
Bond distances			*Bond distances*	
Cd1–N1	2.440(1)	2.437(2)	Cd1–N1	2.465(3)
Cd1–I1	2.939(1)	2.944(2)	Cd1–I1	2.891(1)
Cd1–I1^{i}	2.940(1)	2.912(2)	Cd1–I1^{v}	2.969(1)
Bond angles			*Bond angles*	
Cd1–I1–Cd1^{i}	89.23(1)	87.83(1)	Cd1–I1–Cd1^{v}	89.56(1)
I1–Cd1–I1^{ii}	89.23(1)	87.83(1)	I1–Cd1–I1^{vi}	89.56(1)
I1–Cd1–I1^{iii}	90.78(1)	91.52(1)	I1–Cd1–I1^{vii}	90.44(1)
I1^{ii}–Cd1–I1^{iv}	90.76(1)	92.81(2)		
N1–Cd1–I1	90.09(4)	89.09(5)	N1–Cd1–I1	89.04(6)
N1–Cd1–I1^{ii}	90.39(4)	91.82(5)	N1–Cd1–I1^{vi}	89.82(6)
N1–Cd1–I1^{iii}	89.61(4)	88.17(5)	N1–Cd1–I1^{vii}	90.18(6)
N1–Cd1–I1^{iv}	89.91(4)	90.89(5)	N1–Cd1–I1^{viii}	90.96(6)

Symmetry codes: (*i*) *x*, *y* – 1, *z*; (*ii*) *x*, *y* + 1, *z*; (*iii*) 1 – *x*, *y*, ½ – *z*; (*iv*) 1 – *x*, *y* + 1, ½ – *z*.
(v) *x* + 1, *y*, *z*; (*vi*) *x* – 1, *y*, *z*; (v*ii*) 2 – *x*, 1 – *y*, 1 – *z*; (*viii*) 1 – *x*, 1– *y*, 1 – *z*.

Table S3. Selected bond distances (Å) and angles (˚) for **5:A**.

	**5:A**
Bond distances	
Cd1–N1	2.416(2)
Cd1–Br1	2.756(1)
Cd1–Br1^{i}	2.735(1)
Bond angles	
Cd1–Br1–Cd1^{ii}	90.05(1)
Br1–Cd1–Br1^{i}	90.05(1)
Br1–Cd1–Br1^{iii}	89.95(1)
N1–Cd1–Br1	89.76(5)
N1–Cd1–Br1^{i}	88.96(5)
N1–Cd1–Br1^{iii}	91.04(5)
N1–Cd1–Br1^{iv}	90.24(5)

Symmetry codes: (*i*) *x* – 1, *y*, *z*; (*ii*) *x* + 1, *y*, *z*; (*iii*) 2 – *x*, 1 – *y*, 1 – *z*; (*iv*) 1 – *x*, 1 – *y*, 1 – *z*.

Table S4. Selected bond distances (Å) and angles (˚) for **1:B**.

Bond distances		*Bond*		*Bond*	
Cd1–N1	2.434(3)	Cd2–N5	2.438(3)	Cd3–N9	2.434(3)
Cd1–N3	2.439(3)	Cd2–N7	2.432(3)	Cd3–N11	2.438(3)
Cd1–I1	2.923(1)	Cd2–I3	2.916(1)	Cd3–I1^{i}	2.969(1)
Cd1–I2	2.943(1)	Cd2–I4	2.950(1)	Cd3–I2^{i}	2.971(1)
Cd1–I3	2.987(1)	Cd2–I5	2.988(1)	Cd3–I5	2.923(1)
Cd1–I4	2.957(1)	Cd2–I6	2.956(1)	Cd3–I6	2.942(1)
Bond angles		*Bond angles*		*Bond angles*	
Cd1–I1–Cd3^{ii}	90.09(1)	Cd2–I3–Cd1	89.99(1)	Cd3–I5–Cd2	89.94(1)
Cd1–I2–Cd3^{ii}	89.65(1)	Cd2–I4–Cd1	89.91(1)	Cd3–I6–Cd2	90.21(1)
I1–Cd1–I2	90.80(1)	I3–Cd2–I4	90.81(1)	I5–Cd3–I2^{i}	91.26(2)
I1–Cd1–I3	88.73(2)	I3–Cd2–I5	89.96(2)	I5–Cd3–I6	90.67(1)
I2–Cd1–I4	91.20(2)	I4–Cd2–I6	90.06(2)	I6–Cd3–I1^{i}	88.71(2)
I3–Cd1–I4	89.28(1)	I5–Cd2–I6	89.17(1)	I1^{i}–Cd3–I2^{i}	89.36(1)
N1–Cd1–I1	90.59(7)	N5–Cd2–I3	91.03(8)	N9–Cd3–I1^{i}	89.29(7)
N1–Cd1–I2	91.87(7)	N5–Cd2–I4	91.44(8)	N9–Cd3–I2^{i}	88.58(7)
N1–Cd1–I3	88.53(7)	N5–Cd2–I5	89.19(8)	N9–Cd3–I5	91.03(7)
N1–Cd1–I4	89.68(7)	N5–Cd2–I6	89.59(8)	N9–Cd3–I6	91.56(7)
N3–Cd1–I1	91.51(8)	N7–Cd2–I3	91.43(7)	N11–Cd3–I1^{i}	88.40(7)
N3–Cd1–I2	90.23(8)	N7–Cd2–I4	89.77(7)	N11–Cd3–I2^{i}	89.57(8)
N3–Cd1–I3	89.38(8)	N7–Cd2–I5	89.57(7)	N11–Cd3–I5	91.30(7)
N3–Cd1–I4	88.15(8)	N7–Cd2–I6	87.93(7)	N11–Cd3–I6	90.22(8)

Symmetry codes: (*i*) ½ – *x*, *y* + ½, ½ – *z*; (*ii*) ½ – *x*, *y* – ½, ½ – z.

Table S5. Hydrogen bond distances and angles for **1:A**, **2:A**, **5:A**, **1:B** and **2**.

Compound	C–H···A	*d*(C···A) / Å	*d*(H···A) / Å	∠(C–H···A) / °	R_{HA}*
**1:A**	C3–H3···N3^{ix}	3.399(3)	2.82	120	1.03
	C6–H6···N2	3.455(3)	2.52	167	0.92
**2:A**	C3–H3···N3	3.457(5)	2.77	130	1.01
	C6–H6···N2	3.655(4)	2.78	154	1.01
**5:A**	C3–H3···N3	3.279(4)	2.63	126	0.96
	C6–H6···N2^{i}	3.348(3)	2.76	121	1.00
**1:B**	C3–H3···O2^{ii}	3.312(6)	2.64	128	0.97
	C4–H4···O3	3.097(6)	2.48	123	0.91
	C7–H7···O12^{iii}	3.347(8)	2.80	117	1.03
	C8–H8···O10^{iv}	3.140(6)	2.49	125	0.92
	C11–H11···O4^{ii}	3.338(7)	2.69	126	0.99
	C12–H12···O7	3.106(6)	2.44	127	0.90
	C15–H15···O9^{v}	3.242(7)	2.65	121	0.97
	C16–H16···O6^{iv}	3.082(6)	2.47	122	0.91
	C19–H19···O8^{ii}	3.345(7)	2.73	123	1.00
	C20–H20···O11	3.121(6)	2.45	127	0.90
	C23–H23···O5^{v}	3.289(6)	2.63	127	0.97
	C24–H24···O1^{vi}	3.102(6)	2.48	123	0.91
	C26–H26···N2^{ii}	3.500(5)	2.59	160	0.94
	C29–H29···N6^{ii}	3.496(6)	2.61	156	0.95
	C32–H32···N12^{v}	3.484(5)	2.56	165	0.93
	C35–H35···N10^{ii}	3.474(6)	2.55	164	0.93
	C38–H38···N8^{v}	3.424(6)	2.48	173	0.90
	C41–H41···N4^{vii}	3.469(6)	2.53	170	0.92
**2**	C3–H3···Br^{viii}	3.857(4)	3.02	147	0.99
	C4–H4···N2^{viii}	3.707(4)	2.82	156	1.04

Symmetry codes: (*i*) – *x*, 1 – *y*, – *z*; (*ii*) 1 – *x*, *y*, ½ – *z*; (*iii*) *x* – ½, *y* – ½, *z*; (*iv*) *x* – ½, ½ – *y*, *z* – ½, (*v*) ½ – *x*, ½ – *y*, 1 – *z*; (*vi*) ½ – *x*, *y* + ½, ½ – *z*; (*vii*) *x* + ½, *y* + ½, *z*; (*viii*) 1 – *x*, *y* – ½, 1½ – *z*; (ix) 2 – *x*, *y*, ½ – *z*.

*Normalized value *R*, defined according to Lommerse et al.[10] R_{HA} = *d*(H···A) / (r_H + r_A), where r_H and r_A correspond to van der Waals radii of hydrogen and acceptor atoms (H 1.20 Å, N 1.55 Å, O 1.52 Å, Br 1.85 Å).

Table S6. Halogen bond distances (Å) and angles (˚) for **1:A**, **2:A**, **5:A, 1:B** and **2**.

Compound	C–X···A	d(X···A) / Å	∠(C–X···A) / °	R_{XA}*
**1:A**	C2–I2···I1^{i}	3.686(1)	172.49(5)	0.93
**2:A**	C2–Br1···I1^{i}	3.618(1)	168.33(9)	0.94
**5:A**	C2–Br2···Br1^{ii}	3.578(1)	168.65(8)	0.97
**1:B**	C2–I7···I6^{iii}	3.654(1)	175.53(9)	0.92
	C6–I8···I5^{iv}	3.700(1)	173.28(9)	0.93
	C10–I9···I4^{iii}	3.680(1)	175.12(9)	0.93
	C14–I10···I3^{iv}	3.676(1)	173.57(10)	0.93
	C18–I11···I2^{iii}	3.697(1)	174.31(10)	0.93
	C22–I12···I1^{iv}	3.658(1)	174.96(9)	0.92
**2**	C2–Br1···I1^{v}	3.642(1)	169.06(11)	0.95

Symmetry codes: (*i*) *x*, 1 – *y*, *z* – ½; (*ii*) 2 – *x* , – *y*, 1 – *z*; (*iii*) ½ – *x*, ½ – *y*, – *z*; (*iv*) ½ – *x*, ½ – *y*, 1 – *z;* (v) *x*, *y* + 1, *z*.

*Normalized value *R*, defined according to Lommerse et al.[4] $R_{XA} = d(X\cdots A) / (r_X + r_A)$, where r_X and r_A correspond to van der Waals radii of halogen bond donor and acceptor atoms (Br 1.85 Å, I 1.98 Å).

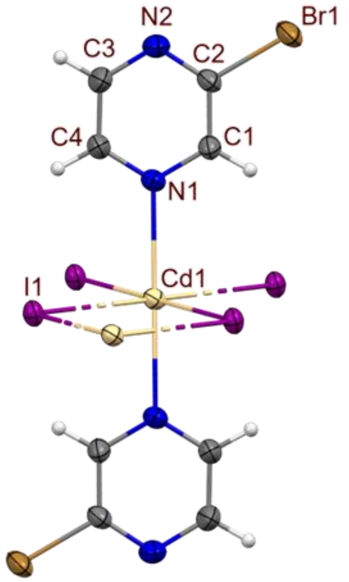

Figure S11. ORTEP-style plot of **2** with a partial labeling scheme. Thermal ellipsoids are drawn at a 50% probability level at 200(2) K.

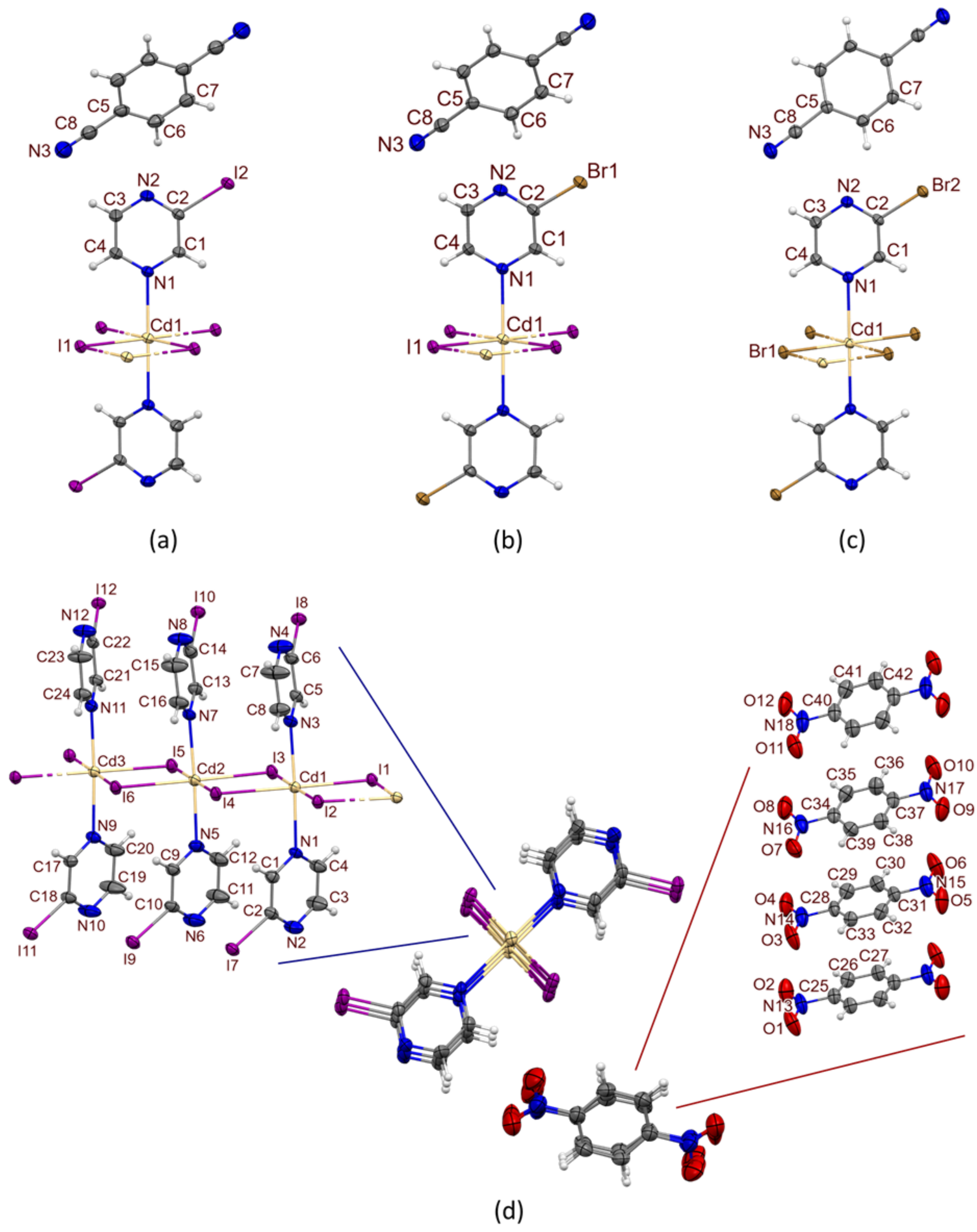

Figure S12. ORTEP-style plot of **1:A** (a), **2:A** (b), **5:A** (c) and **1:B** (d) with a partial labelling scheme. Thermal ellipsoids are drawn at a 50% probability level at 170(1) K.

5. Computational studies / Molecular electrostatic potential maps

The neighboring polymeric layers in the parents (**1**–**9**) are mutually linked *via* four HBs per polymeric unit, two C–H_{ortho}···N and two C–H_{meta}···X', while in the co-crystals (**1:A**, **2:A**, **5:A**), the co-formers intervened between the neighboring 2-D layers, and are linked with their polymeric neighbors *via* two C_A–H_A…N_{CP} and two C_{CP}–H_{meta}…N_A. The same set of four HBs per polymeric unit was formed in all co-crystals with **A** for which the crystal structure was determined.

Only hydrogen bonds observed in the crystal structures of both pure forms (CPs and co-formers) and co-crystals that formed (**1:A**, **2:A**, **5:A**) were taken into account (ΣvdW + 0.1 Å), and the same set of hydrogen bonds was presumed to form in all other co-crystals for which the crystal structure was not determined (**3:A**, **6:A**) or did not form under the examined experimental conditions (**4:A**, **7:A**, **8:A**, **9:A**).

The parameters α_i and β_i were calculated using maxima and minima on the MEP surfaces, respectively. The interaction energies were derived by summing their products for each hydrogen-bond interaction realized in the crystal structure:

$$\alpha_i = 0.0000162\ MEP_{max}^2 + 0.00962\ MEP_{max}$$

$$\beta_i = 0.000146\ MEP_{min}^2 - 0.00930\ MEP_{min}$$

$$E = -\sum_i \alpha_i \beta_i$$

The probability of co-crystal formation was estimated on the difference in the surface site interaction point pairing energies between the co-crystal (E_{cc}) and the two pure forms (the parent coordination polymer, E_{CP}, and co-former, E_{cf}) in a 1:1 stoichiometric ratio:

$$\Delta E = E_{cc} - (E_{CP} + E_{cf})$$

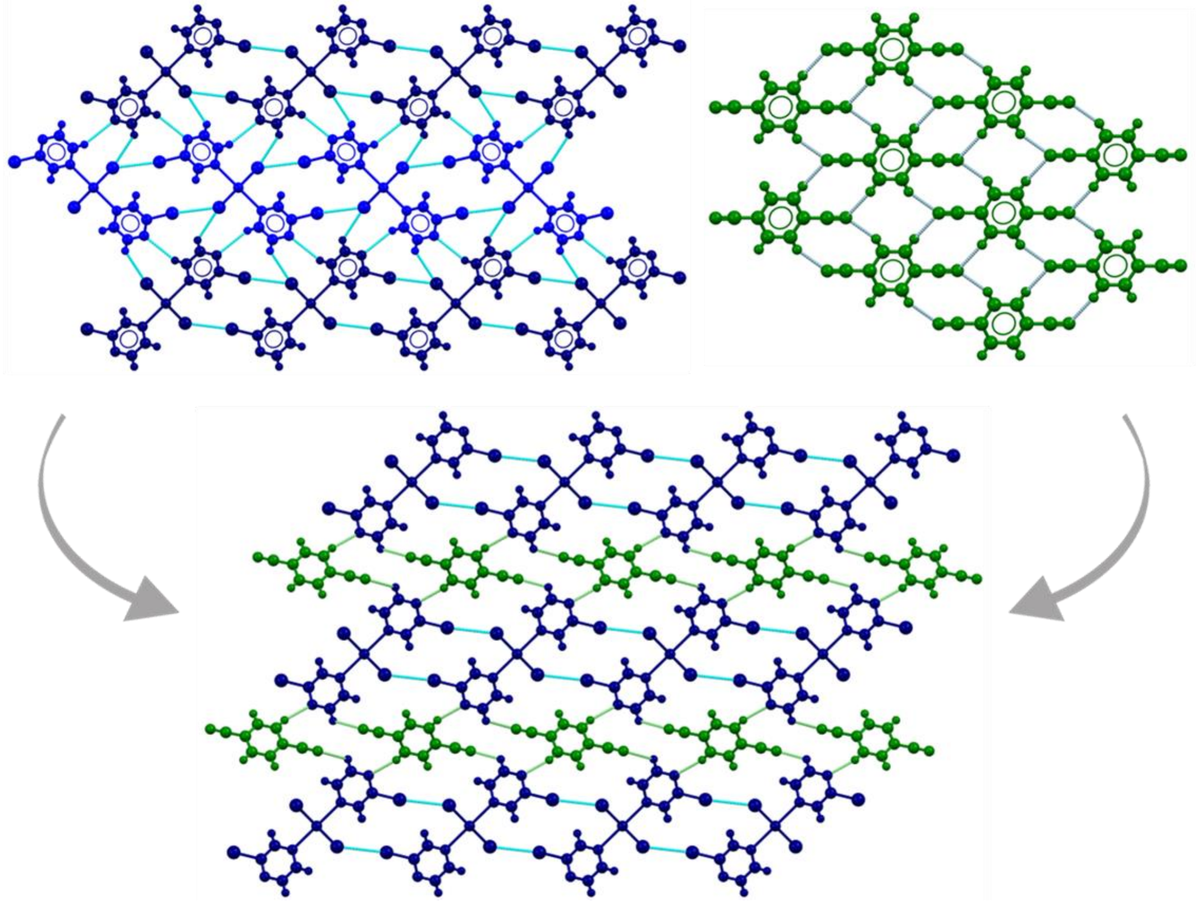

Figure S13. Crystal packing of the starting coordination polymer, *refcode*: **YIKJEK**, co-former, *refcode*: **TEPNIT13**, and resulting co-crystal **1:A**.

	COORDINATION POLYMER										E_{CP}
	H_{meta}	$\alpha(H_{meta})$	H_{ortho}	$\alpha(H_{ortho})$	N	β(N)	X′	β(X′)	$\alpha(H_{ortho})\cdot\beta(N)$	$\alpha(H_{meta})\cdot\beta(X')$	$-2\sum\alpha_i\cdot\beta_i$
**1**	144	1.72120	97	1.08557	−131	3.72381	−57	1.00445	−4.04244	−1.72887	−11.54261
**2**	157	1.90965	112	1.28065	−125	3.44375	−45	0.71415	−4.41025	−1.36378	−11.54805
**3**	163	1.99848	118	1.36073	−122	3.30766	−39	0.58477	−4.50083	−1.16864	−11.33895
**4**	149	1.79304	98	1.09834	−128	3.58246	−95	2.20115	−3.93478	−3.94674	−15.76304
**5**	162	1.98359	114	1.30722	−121	3.26289	−84	1.81138	−4.26529	−3.59303	−15.71665
**6**	170	2.10358	120	1.38768	−118	3.13030	−78	1.61366	−4.34386	−3.39447	−15.47666
**7**	147	1.76421	95	1.06011	−131	3.72381	−113	2.91517	−3.94763	−5.14297	−18.18118
**8**	161	1.96874	112	1.28065	−124	3.39810	−105	2.58615	−4.35178	−5.09146	−18.88648
**9**	168	2.07339	119	1.37419	−121	3.26289	−102	2.46758	−4.48382	−5.11626	−19.20016

CO-FORMER					E_{cf}
	H_A	$\alpha(H_A)$	N_A	$\beta(N_A)$	$-2\sum\alpha_i\cdot\beta_i$
**A**	168	2.07339	−155	4.94915	−20.52302

CO-CRYSTAL											E_{CC}
	H_{meta}	$\alpha(H_{meta})$	N_{CP}	$\beta(N_{CP})$	H_A	$\alpha(H_A)$	N_A	$\beta(N_A)$	$\alpha(H_{meta})\cdot\beta(N_A)$	$\alpha(H_A)\cdot\beta(N_{CP})$	$-2\sum\alpha_i\cdot\beta_i$
**1:A**	144	1.72120	−131	3.72381	168	2.07339	−155	4.94915	−8.51849	−7.72090	−32.47878
**2:A**	157	1.90965	−125	3.44375	168	2.07339	−155	4.94915	−9.45116	−7.14023	−33.18279
**3:A**	163	1.99848	−122	3.30766	168	2.07339	−155	4.94915	−9.89077	−6.85807	−33.49768
**4:A**	149	1.79304	−128	3.58246	168	2.07339	−155	4.94915	−8.87401	−7.42784	−32.60369
**5:A**	162	1.98359	−121	3.26289	168	2.07339	−155	4.94915	−9.81710	−6.76523	−33.16466
**6:A**	170	2.10358	−118	3.13030	168	2.07339	−155	4.94915	−10.41093	−6.49034	−33.80254
**7:A**	147	1.76421	−131	3.72381	168	2.07339	−155	4.94915	−8.73132	−7.72090	−32.90443
**8:A**	161	1.96874	−124	3.39810	168	2.07339	−155	4.94915	−9.74359	−7.04557	−33.57833
**9:A**	168	2.07339	−121	3.26289	168	2.07339	−155	4.94915	−10.26151	−6.76523	−34.05349

6. Thermogravimetric analysis (TGA) and Differential Scanning Calorimetry (DSC)

Thermogravimetric analysis was performed using a simultaneous TGA-DTA analyzer Mettler-Toledo TGA/DSC3+. The powder samples (**2:A**, **3:A, 5:A**, **6:A**) were placed in alumina pans (70 µL), heated in flowing nitrogen (50 mL min^{-1}) from room temperature up to 600 °C at a rate of 10 °C min^{-1}. Data collection and analysis were performed using the program package STARe Software 16.20 MettlerToledo GmbH, 2015.[11]

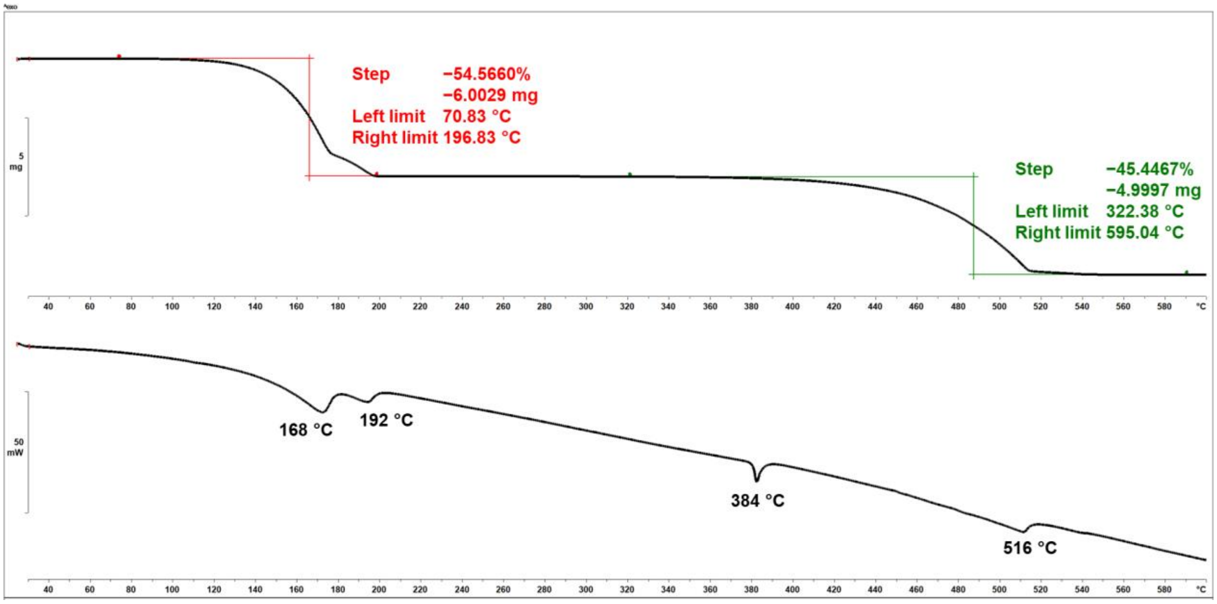

Figure S14. TGA curve (up) and DSC curve (down) observed for **2:A**.

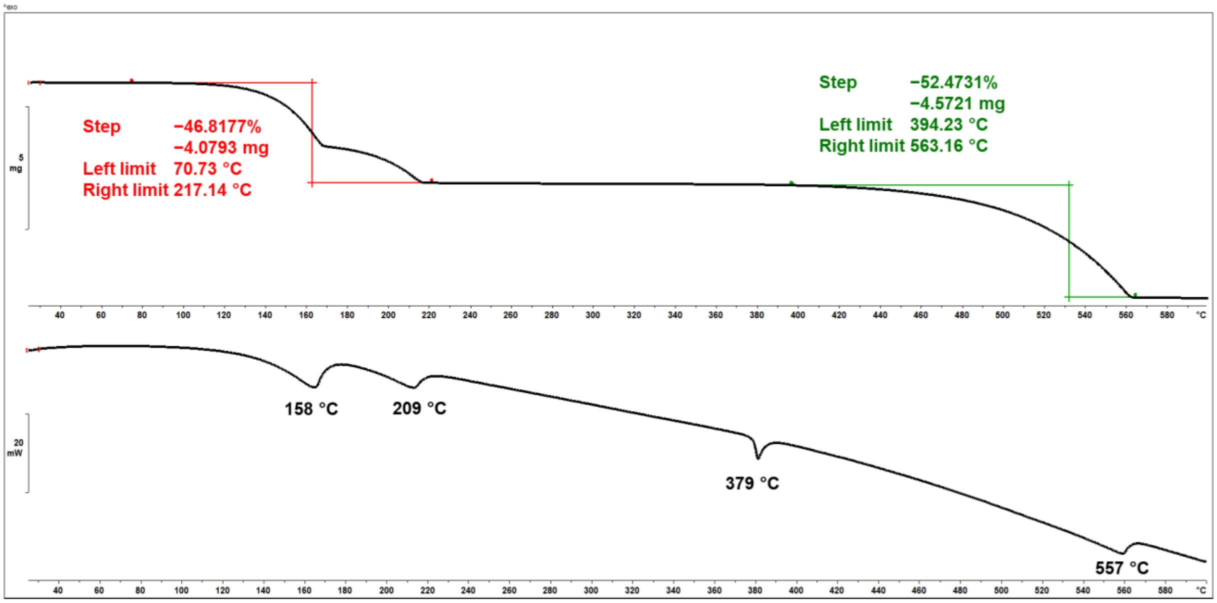

Figure S15. TGA curve (up) and DSC curve (down) observed for **3:A**.

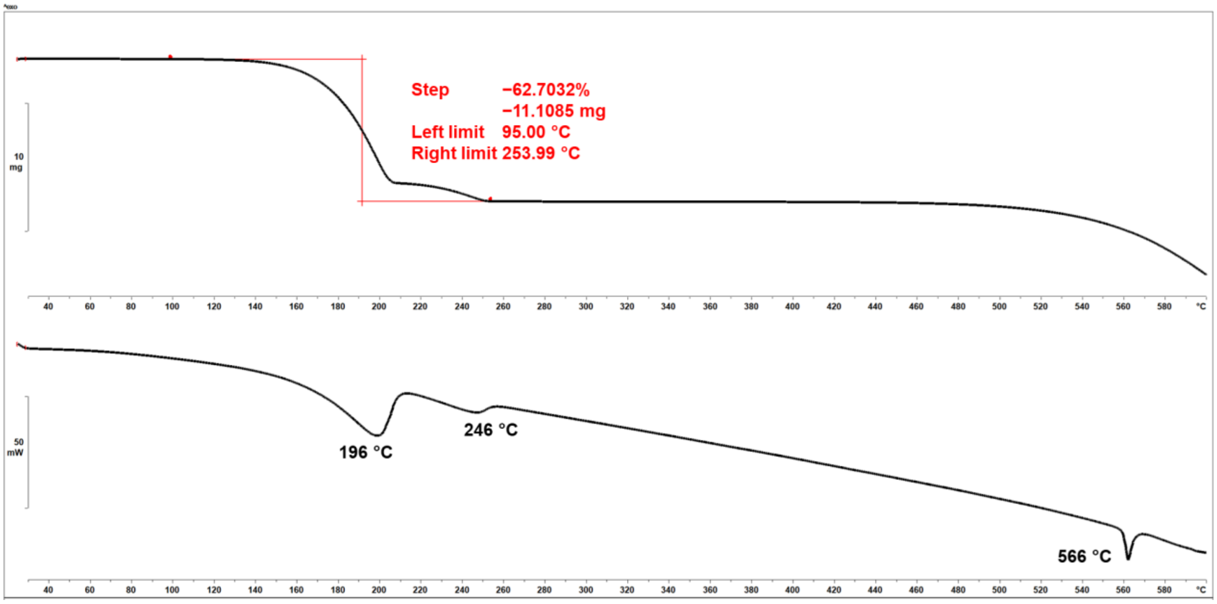

Figure S16. TGA curve (up) and DSC curve (down) observed for **5:A**.

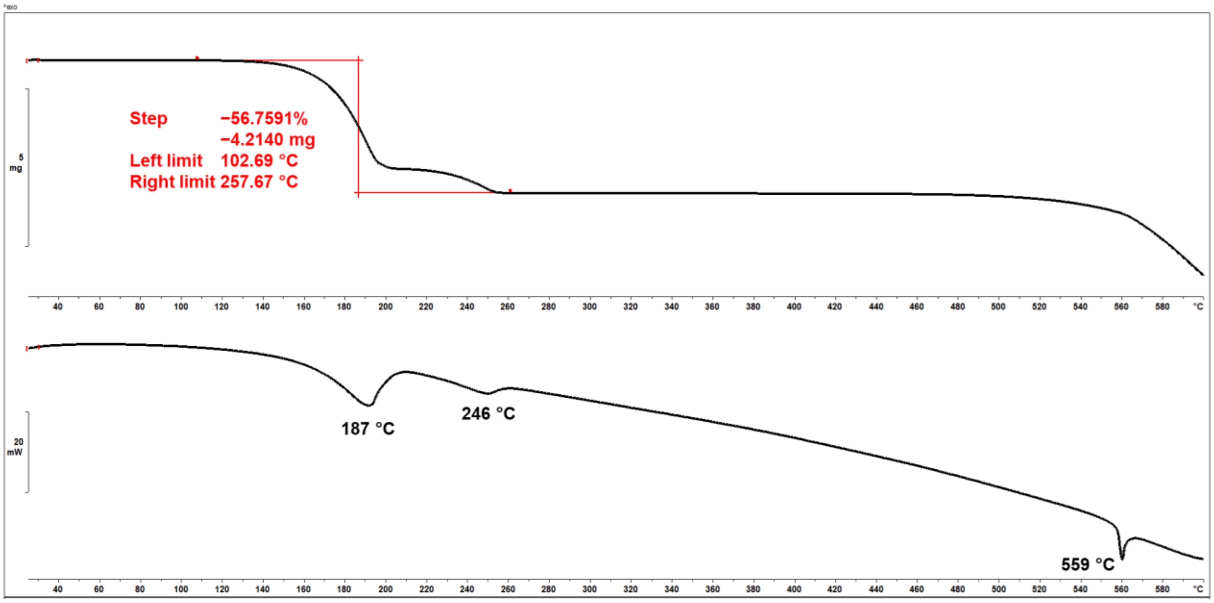

Figure S17. TGA curve (up) and DSC curve (down) observed for **6:A**.

7. Infrared Spectroscopy (FT-IR)

Vibrational analysis in the infrared region was performed using *Attenuated total reflection* (ATR) sampling technique on the *PerkinElmer Spectrum Two* spectrometer with an *Diamond UATR* accessory. The small amount of powder samples (**1:A**, **1:B**, **2:A**, **3:A**, **5:A** and **6:A**) were placed on the diamant reflexion element, and data collection was performed in the wavenumber range of 4000 cm^{-1} to 400 cm^{-1}, with the resolution of 4 cm^{-1}. Data collection and analysis were performed using the program package *Spectrum*, version 10.4.2.[12]

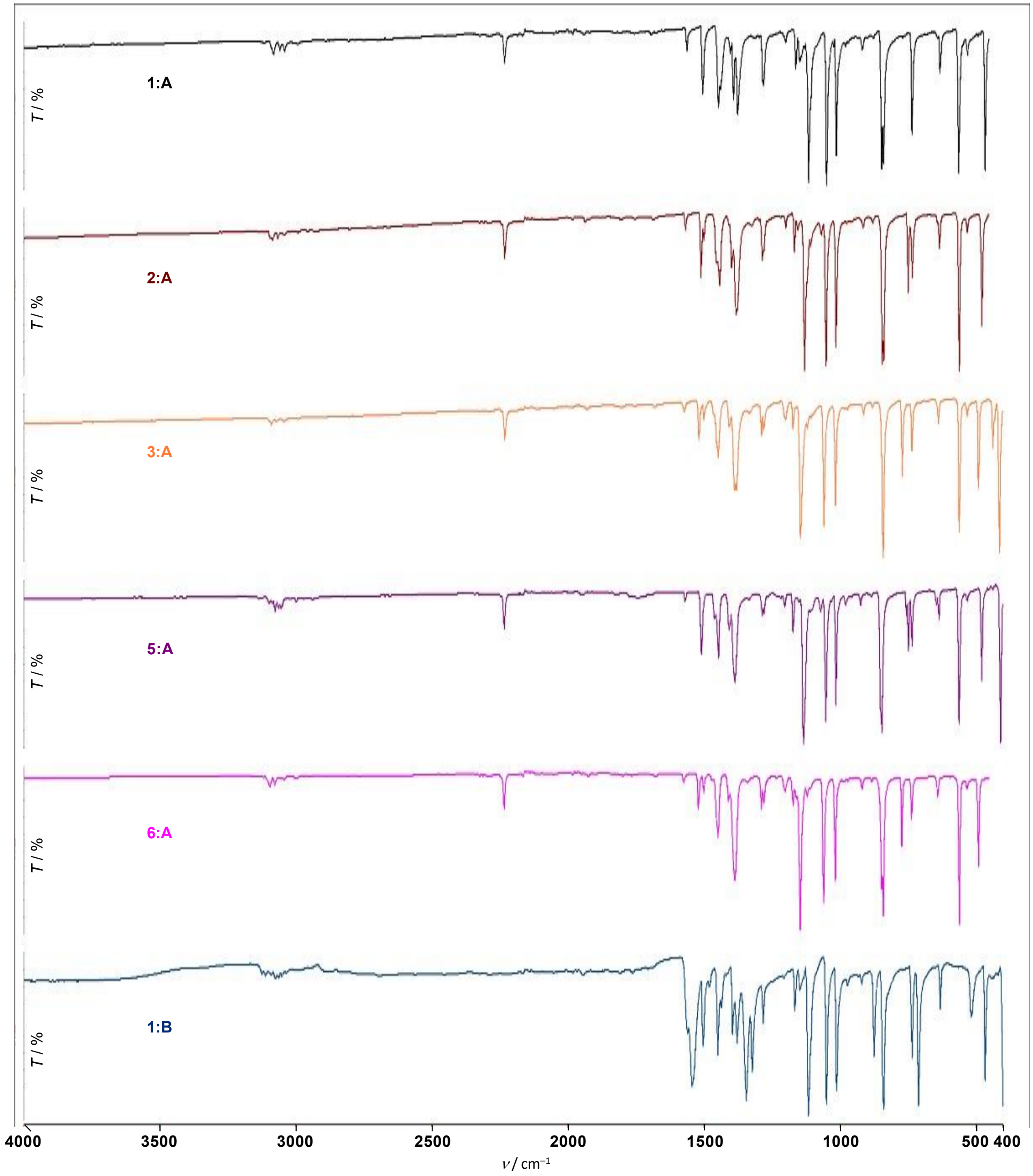

Figure S18. FT-IR (ATR) spectra of resulting co-crystals: **1:A** (**black**), **2:A** (**brown**), **3:A** (**orange**), **5:A** (**purple**), **6:A** (**pink**) and **1:B** (**blue**).

References

1. Groom, C. R.; Bruno, I. J.; Lightfoot M. P.; Ward, S. C. The Cambridge Structural Database. *Acta Cryst.*, **2016**, *B72*, 171–179.
2. Data Viewer, version 1.9a, PANalytical B. V., Almelo, The Netherlands.
3. CrysAlisPRO, Oxford Diffraction/Agilent Technologies UK Ltd, Yarnton, England.
4. Sheldrick, G. M. SHELXT-Integrated space-group and crystal-structure determination. *Acta Crystallogr*. **2015**, *A71*, 3–8.
5. Sheldrick, G. M. Crystal structure refinement with SHELXL, *Acta Cryst.* **2015**, *C71*, 3–8.
6. Sheldrick, G. M. A short history of SHELX, *Acta Cryst.* **2008**, *A64*, 112–122.
7. a) Le Page, Y. Computer derivation of the symmetry elements implied in a structure description, *J. Appl. Cryst.* **1987**, 20, 264–269.
   b) Le Page, Y. MISSYM1.1 – a flexible new release, *J. Appl. Cryst.* **1988**, 21, 983–984.
8. Spek, A. L. checkCIF validation ALERTS: what they mean and how to respond, *Acta Cryst.* **2020**, *E76*, 1–11.
9. a) Macrae, C. F.; Sovago, I.; Cottrell, S. J.; Galek, P. T. A.; McCabe, P.; Pidcock, E.; Platings, M.; Shields, G. P.; Stevens, J. S.; Towler M.; Wood, P. A. Mercury 4.0: from visualization to analysis, design and prediction, *J. Appl. Cryst.,* **2020**, 53, 226–235.
   b) Macrae, C. F.; Bruno, I. J.; Chisholm, J. A.; Edgington, P. R.; McCabe, P.; Pidcock, E.; Rodriguez-Monge, L.; Taylor, R.; van de Streek, J.; Wood, P. A. New features for the visualization and investigation of crystal structures. *J. Appl. Crystallogr*. **2008**, *41*, 466–470.
10. Lommerse, J. P. M.; Stone, A. J.; Taylor, R.; Allen, F. H. The Nature and Geometry of Intermolecular Interactions between Halogens and Oxygen or Nitrogen, *J. Am. Chem. Soc.*, **1996**, *118*, 3108–3116.
11. STAReSoftware 16.20, MettlerToledoGmbH, **2006**.
12. Perkinelmer spectrum, 2014. URL http://perkinelmer.com. PerkinElmer Ltd, UK.
